# Modulation of Glial Responses by Furanocembranolides: Leptolide Diminishes Microglial Inflammation in Vitro and Ameliorates Gliosis In Vivo in a Mouse Model of Obesity and Insulin Resistance

**DOI:** 10.3390/md18080378

**Published:** 2020-07-22

**Authors:** Miriam Corraliza-Gómez, Amalia B. Gallardo, Ana R. Díaz-Marrero, José M. de la Rosa, Luis D’Croz, José Darias, Eduardo Arranz, Irene Cózar-Castellano, María D. Ganfornina, Mercedes Cueto

**Affiliations:** 1 Instituto de Biología y Genética Molecular, Universidad de Valladolid-CSIC, 47003 Valladolid, Spain; miriamcorraliza@gmail.com (M.C.-G.); earranz@uva.es (E.A.); cozarirene@gmail.com (I.C.-C.); 2 Instituto de Productos Naturales y Agrobiología (IPNA-CSIC), Avenida Astrofísico F. Sánchez, 3, 38206 La Laguna, Tenerife, Spain; amalia.gallardo@umag.cl (A.B.G.); adiazmar@ull.edu.es (A.R.D.-M.); jmrosa@ull.edu.es (J.M.d.l.R.); dariasjose4@gmail.com (J.D.); 3 Departamento de Ciencias y Recursos Naturales, Facultad de Ciencias, Universidad de Magallanes, Avenida Bulnes 01855, Punta Arenas, Chile; 4 Departamento de Biología Marina y Limnología, Universidad de Panamá, Panama 3366, Panama; dcrozlc@gmail.com; 5 Smithsonian Tropical Research Institute, STRI, Box 0843-03092 Balboa, Panama; 6 Centro de Investigación Biomédica en Red de Diabetes y Enfermedades Metabólicas Asociadas (CIBERDEM), 28029 Madrid, Spain

**Keywords:** *Leptogorgia*, furanocembranolides, leptolide, microglia, astrocytes, gliosis, anti-inflammatory compounds, bioactive natural products, drug discovery, high-fat diet

## Abstract

Neurodegenerative diseases are age-related disorders caused by progressive neuronal death in different regions of the nervous system. Neuroinflammation, modulated by glial cells, is a crucial event during the neurodegenerative process; consequently, there is an urgency to find new therapeutic products with anti-glioinflammatory properties. Five new furanocembranolides (**1**−**5**), along with leptolide, were isolated from two different extracts of *Leptogorgia* sp., and compound **6** was obtained from chemical transformation of leptolide. Their structures were determined based on spectroscopic evidence. These seven furanocembranolides were screened in vitro by measuring their ability to modulate interleukin-1β (IL-1β) production by microglial BV2 cells after LPS (lipopolysaccharide) stimulation. Leptolide and compounds **3**, **4** and **6** exhibited clear anti-inflammatory effects on microglial cells, while compound **2** presented a pro-inflammatory outcome. The in vitro results prompted us to assess anti-glioinflammatory effects of leptolide in vivo in a high-fat diet-induced obese mouse model. Interestingly, leptolide treatment ameliorated both microgliosis and astrogliosis in this animal model. Taken together, our results reveal a promising direct biological effect of furanocembranolides on microglial cells as bioactive anti-inflammatory molecules. Among them, leptolide provides us a feasible therapeutic approach to treat neuroinflammation concomitant with metabolic impairment.

## 1. Introduction

It has been estimated that, by the year 2050, 1 in 6 people in the world will be over the age of 65 [1]. In other words, over the next three decades, the global number of people over the age of 60 is predicted to more than double, reaching over 1.5 billion people worldwide in 2050 [1]. Due to the increase in life expectancy, age-related diseases are expected to rise in incidence. One of the most prominent groups of these diseases are neurodegenerative disorders, characterized by the loss of neurons within the brain and/or spinal cord [2]. Consequently, the prevalence of neurodegenerative diseases is also expected to rise dramatically in the next few decades, with the resultant additional socioeconomic burden. Therefore, drugs to slow down or stop the neurodegenerative process are urgently needed [3].

Common factors that are potential mediators of the neurodegenerative process include increased oxidative stress, impaired energy metabolism, lysosomal dysfunction, protein aggregation, inflammation, excitoxicity, necrosis and/or apoptosis [4]. The “neurocentric” approach to treat neurodegenerative diseases has dominated the research focus for many years, with great efforts to develop drugs to avoid neuronal death. Nevertheless, with the increasing knowledge about glial cells, it is becoming evident that controlling inflammation within the brain requires the study of glia. Hence, an expansion from an exclusively “neurocentric” to a “gliocentric” focus would improve the prospects of improved future therapeutic strategies against neurodegeneration.

The brain was considered an immunologically privileged organ until a few years ago, but nowadays, it is accepted that both microglia and astrocytes are involved in modulating inflammation within the central nervous system (CNS). Microglia are the resident immune cells of the CNS and play a crucial role in brain homeostasis during aging. In fact, microglial cells are the most important players in the communication route between the CNS and the peripheral immune system [5,6]. Consequently, microglial cells are highly sensitive to small changes in the brain microenvironment in order to switch their functions to maintain brain equilibrium. Similarly to peripheral macrophages, microglia have been often classified into either “classically” or “alternatively” activated phenotypes [7,8]. However, this categorization is oversimplified given their high degree of diversity and plasticity [9]. During recent years, this concept is being replaced by a spectrum of activation patterns of microglial cells, for which the extremes are pro- and anti- inflammatory phenotypes [10]. Mounting evidence suggests that certain situations such as infection or aging can alter the threshold for microglial activation. This has a profound impact on microglial phenotypes, switching towards a pro-inflammatory state that triggers maladaptive responses that contribute to the onset and progression of some CNS diseases [11].

While microglia have their own inflammatory phenotype axis, they also modulate pro-inflammatory and neurotoxic activities in astrocytes in different ways. Classically activated microglia promote astrocyte differentiation into a reactive state by secreting pro-inflammatory cytokines such as IL-1β and tumor necrosis factor alpha (TNF-α) [12]. Moreover, in the injured brain, astrocytes are reported to act as controllers to rapidly restrain microglial activation, thus avoiding exacerbated responses to injuries [13]. Such evidence suggests there is a tightly regulated equilibrium between glial cells that regulates local immune reactions within the brain.

Octocorals of the genus *Leptogorgia* produce furanocembranolides, highly oxygenated diterpenes into which a γ-lactone subunit is embedded. Marine furanocembranolides can be divided into four different classes: those in which the substituent at the C-4 position is a CH_3_ (class A), those with CHO (class B), those with COOH (class C) and those with COOCH_3_ (class D) [14,15]. Although the pharmacological potential of this family of diterpenes is largely unexplored, a few examples of anti-inflammatory activity have been reported. Among them, sinulacembranolide A and crassarine H were found to significantly reduce the levels of the pro-inflammatory protein iNOS (inducible nitric oxide synthase) relative to control cells stimulated with LPS (lipopolysaccharide) only [16,17].

Increasing evidence based on epidemiological studies and preclinical models suggests that a strong link exists between metabolic dysfunction and neurodegeneration. In fact, impaired energy metabolism is a hallmark of neurodegenerative disorders [18,19]. Interestingly, diabetes mellitus patients are predisposed to suffer from dementia. Some of the known mechanisms leading to this complication are changes in the vasculature structure and function, glucose metabolism dysregulation, insulin signalling impairment, inflammation, and modifications in processing and degradation of amyloid beta and hyperphosphorylated tau [20,21,22]. Regarding the family of furanocembranolides, leptolide has been shown to act as an insulin sensitizer, enhancing insulin signalling and sensitivity in the liver and muscle of high-fat diet-induced obese mice [23]. Leptolide reduced weight gain in these mice in parallel with decreased circulating pro-inflammatory cytokines and leptin. These results led us to hypothesize that furanocembranolides, due to their beneficial effects on metabolism and systemic inflammation, could also have beneficial effects on the CNS and potentially counteract the complications developed in a preclinical model of high-fat diet-induced obesity and insulin resistance.

**Figure 1:**
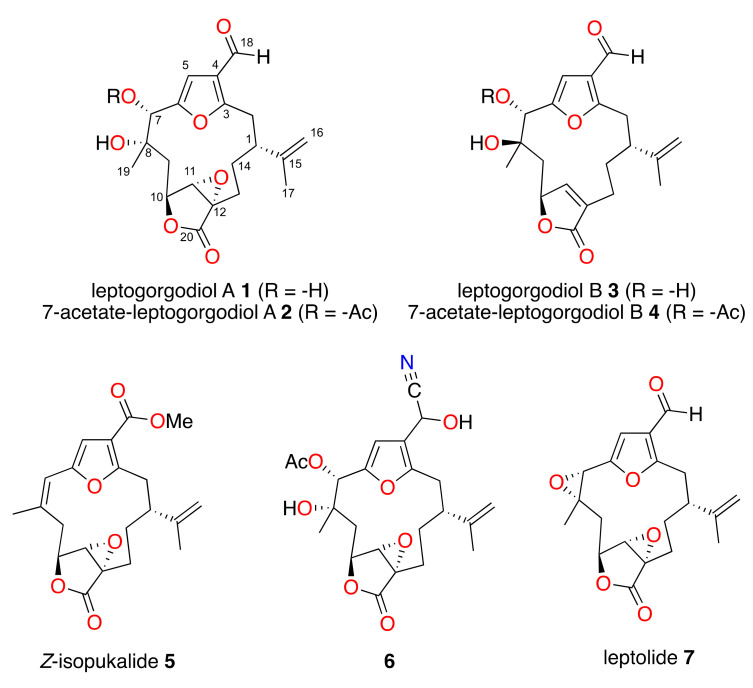
Novel furanocembranolides (**1**−**6)** and leptolide (**7**).

These antecedents prompted us to re-examine two extracts of *Leptogorgia* sp., from which new furanocembranolides **1–5** were obtained, together with the previously known leptolide (**7**) [14]. Furthermore, we treated leptolide to obtain compound **6** (Figure 1) to assess a new furanocembranolide with different substituents at position C-4.

We assayed the potential anti-inflammatory effects of seven members of the furanocembranoid family in vitro by measuring their capacity to diminish IL-1β mRNA production by microglial BV2 cells after LPS (lipopolysaccharide) stimulation. Among the compounds that showed anti-inflammatory effects in vitro, we chose leptolide to appraise its anti-glioinflammatory potential in vivo, treating high-fat diet-induced obese mice with leptolide and quantifying gliosis. Taken together, our results reveal a promising effect of furanocembranolides as bioactive anti-inflammatory molecules, and we propose leptolide as a feasible therapeutic approach to reduce the neuroinflammation concomitant with metabolic impairment.

## 2. Results

### 2.1. Characterization of Novel Furanocembranolides

Leptogorgodiol A (**1**), obtained as an oil, showed a peak at *m/z* [M]^+^ 376 in the electron impact mass spectrometry (EIMS) spectrum corresponding to the molecular formula C_20_H_24_O_7_ (High resolution electron impact mass spectrometry(HREIMS)) (*m/z* 376.1514 [M]^+^ calcd. for C_20_H_24_O_7_ 376.1522). The diterpenic nature of **1** is confirmed by ^13^C nuclear magnetic resonance (NMR) and heteronuclear single quantum correlation (HSQC) spectra indicative of seven quaternary carbons, six methines, five methylenes and two methyls (Table 1). Absorptions for a hydroxyl group at 3478 cm^−1^ and carbonyl groups at 1774 and 1672 cm^−1^ were observed in the infrared (IR) spectrum.

Significant ^1^H and ^13^C NMR signals were an aldehyde group (δ_H-18_ 9.89 (1H, s) and δ_C-18_ 184.7), a trisubstituted furane ring (δ_H-5_ 6.69 (1H, s), δ_C-5_ 106.4, δ_C-3_ 162.4, δ_C-6_ 155.2 and δ_C-4_ 123.2), a α,β-epoxy-γ-lactone ring (δ_H-11_ 3.70 (1H, s), δ_C-11_ 63.0, δ_C-20_ 172.5, δ_H-10_ 4.85 (1H, dd, *J* = 5.0, 11.0 Hz), δ_C-10_ 74.5 and δ_C-12_ 61.0) and an isopropenyl group (δ_C-15_ 144.9, δ_H-16a_ 4.97 (1H, dd, *J* = 1.6, 1.6 Hz), δ_H-16b_ 5.10 (1H, *s*), δ_C-16_ 114.1, δ_H-17_ 1.77 (3H, s) and δ_C-17_ 18.8). Information obtained from homonuclear correlation spectroscopy (COSY), HSQC and heteronuclear multiple-bond correlation spectroscopy (HMBC) experiments unambiguously established the planar structure of compound **1** as a furanocembranolide that contains a C-18 oxidized to aldehyde, a vicinal diol at C-7C-8, a C-10−C-20 α,β-epoxy-γ-lactone moiety and an isopropenyl group at C-1.

**Figure 2:**
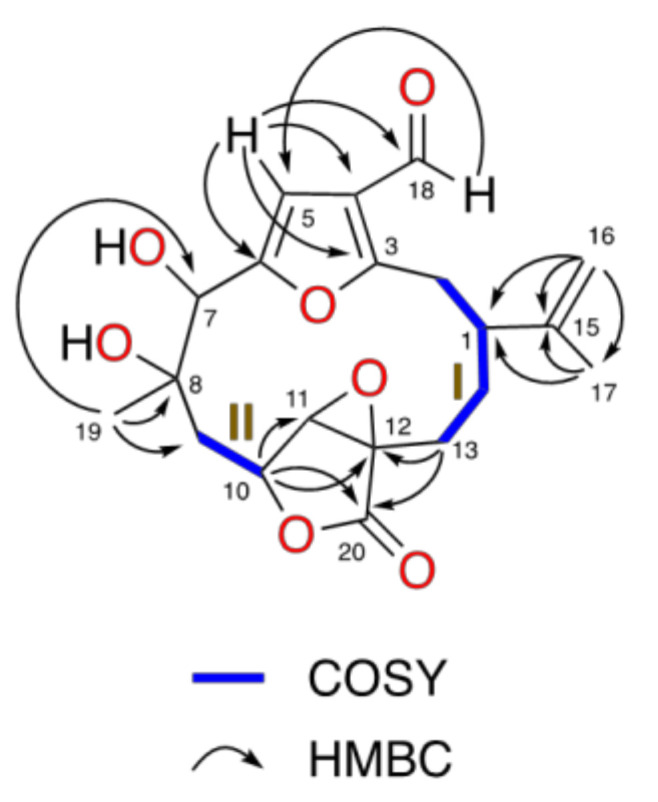
^1^H−^1^H-COSY (—), HMBC (→) correlations of **1**.

The ^1^H–^1^H-COSY experiment confirmed two spin systems: H_2_-2–H_2_-13 (fragment **I**) and H_2_-9–H-10 (fragment **II**) (Figure 2). The HMBC correlations H_2_-16/C-17, C-1 and H_3_-17/C-16, C-15, C-1 locate an isopropenyl group at C-1 of fragment **I**, while the correlations H_3_-19/C-7, C-8 and C-9 permits a lengthening of fragment **II** by connecting a vicinal dihydroxyl moiety to C-9. The north end of both fragments **II** and **I** are connected together by insertion of a furane ring, which is in agreement with the HMBC correlations H-5/C-3, C-4, C-6 and H_2_-2 with C-3. The southern ends of fragments **I** and **II** between C-13 and C-10 are joined to an epoxy lactone, as deduced from the HMBC correlations H-10/C-11, C-20; H_2_-13/C-20, C-11, C-12; and H_2_-14 with C-12. Therefore, the structure of compound **1** was established, showing nine degrees of unsaturation as shown in Figure 2.

7-Acetate-leptogorgodiol A (**2**) was obtained as an oil. Its EIMS spectrum showed a molecular ion at *m/z* [M]^+^ 418 that corresponds to the molecular formula C_22_H_26_O_8_ (HREIMS) (*m*/*z* 418.1619 [M]^+^; calcd. for C_22_H_26_O_8_ 418.1628). The ^13^C NMR spectrum and correlations observed in the HSQC spectrum indicated eight quaternary carbons, six methines, five methylenes and three methyls (Table 1).

^1^H and ^13^C NMR data resemble those of compound **1**. The main differences lie in the presence of an acetoxy group (δ_H_ 2.12 (3H, *s*,), δ_C_ 21.0 and δ_C_ 170.1) and the downfield chemical shift of H-7 (δ_H-7_ 6.21 (1H, *s*)). These shift values suggested that compound **2** is the C-7 acetyl derivative of **1**. Thus, the structure of **2** with ten degrees of unsaturation was established as shown in Figure 1 by COSY, HSQC and HMBC experiments.

Leptogorgodiol B (**3**) was obtained as an oil. The EIMS spectrum showed a peak at *m/z* 360 [M]^+^, corresponding to the molecular formula C_20_H_24_O_6_ (HREIMS) (*m/z* 360.1561 [M]^+^, calcd. for C_20_H_24_O_6_ 360.1573). These data agree with the ^13^C NMR spectrum and the HSQC correlations which confirmed seven quaternary carbons, six methines, five methylenes and two methyls. The degree of unsaturation given by the ^13^C NMR data (Table 2) indicated that **3** must be a tricyclic compound.

Comparison of the ^1^H and ^13^C NMR data for compounds **1** and **3** indicates that, in compound **3**, there is an α,β-unsaturated-γ-lactone ring (δ_H-11_ 5.81 (1H, *s*), δ_C-11_ 148.7, δ_C-12_ 133.6, δ_C-20_ 173.5 and δ_C-10_ 78.8) instead of the α,β-epoxy-γ-lactone ring of compound **1**. This is in agreement with the molecular formulas of compounds **1** and **3** that diverge by 16 amu of oxygen. Therefore, the structure of **3**, containing nine degrees of unsaturation, was determined as shown in Figure 1 by COSY, HSQC and HMBC experiments.

7-Acetate-leptogorgodiol B (**4**), obtained as an oil, showed a peak at *m/z* 402 [M]^+^ in the EIMS spectrum, which corresponds to the formula C_22_H_26_O_7_ (HREIMS) (*m/z* 402.1688 [M]^+^; calcd. for C_22_H_26_O_7_, 402.1679). The HSQC correlations and the signals observed in the ^13^C NMR spectrum indicate eight quaternary carbons, six methines, five methylenes and three methyls.

^1^H and ^13^C NMR data (Table 2) resemble those of **3**. The principal differences lie in the presence of an acetoxy group (δ_H_ 2.16 (3H, *s*,), δ_C_ 21.1 and δ_C_ 169.5) and the downfield chemical shift of H-7 (δ_H-7_ 5.59 (1H, *s*)). Together with the HMBC correlations of H-7 with the carbonyl carbon in the acetoxy group, these shift values indicated that compound **4** is the C-7 acetyl derivative of **3**. The planar structure of **4** (Figure 1), with ten degrees of unsaturation, was further corroborated as shown in Figure 1 by COSY, HSQC and HMBC experiments.

*Z*-isopukalide (**5**) was obtained as an oil. The EIMS spectrum showed a peak at *m/z* 372 [M]^+^, which agrees with the molecular formula C_21_H_24_O_6_ (HREIMS) (*m/z* 372.1575 [M]^+^, calcd. for C_21_H_24_O_6_, 372.1573). These data are consistent with the ^13^C NMR spectrum and the correlations in the HSQC experiment indicating eight quaternary carbons, five methines, five methylenes and three methyls, one of them a methyl ester (Table 3).

In addition to the ^1^H and ^13^C NMR data supporting an isopropenyl group, a furan ring and an α,β-epoxy-γ-lactone, other key signals were detected for a methyl ester group (δ_H-21_ 3.82 (3H, s), δ_C-21_ 51.7 and δ_C-18_ 164.3). Therefore, the 21 carbon atoms in the molecular formula suggest that the characteristic isoprenic methyl group C-18 found in regular furanocembranolides is oxidized to a methyl ester, placing compound **5** in class D [14]. Its planar structure with ten degrees of unsaturation was further corroborated to be as shown in Figure 1 by COSY, HSQC and HMBC experiments.

Marine furanocembranolides can be divided into four classes according to the substituent at position C-4, those bearing CH_3_ (class A), CHO (class B), COOH (class C) or COOCH_3_ (class D). Our previous studies of octocorals from the coasts of Panama allowed us to isolate furanocembranolides in classes A, B and D [14,15]. We also showed that furanocembranoids belonging to classes A, B and D improve pancreatic β-cell proliferation [23,24,25]. In order to obtain new furanocembranolides with different substituents at the C-4, we treated leptolide with acetic acid and NaCN and later with MnO_2_ (see the Experimental Section). After purification by flash chromatography, a mixture of two epimers at C-18 of the cyano derivative **6** was obtained, together with an acetoxy derivative for which the MS spectrometry and NMR data coincide with those of compound **2**.

The EIMS spectrum for the mixture of the epimers of 6 shows a peak at m/z 446 [M + H]^+^, which corresponds to the molecular formula C_23_H_28_NO_8_ (HREIMS) (m/z 446.1810 [M]^+^, calcd. for C_23_H_28_NO_8_, 446.1815). Some of the ^1^H and ^13^C NMR signals of 6 appear duplicated due to the presence of the two epimers.

The incorporation of the cyano and acetoxy groups in **6** is corroborated by the ^13^C NMR spectrum, and the HSQC correlations are indicative of nine quaternary carbons (one cyano group and two carbonyls), six methines, five methylenes and three methyls. The presence of the acetoxy group at C-7 was established by HMBC correlation of C-21 (CO) with H-7, and the cyano at C-18 was established by those correlations of H-18 with C-5 and C-23 (CN).

### 2.2. Relative Configuration of Compounds 1–6

**Figure 3:**
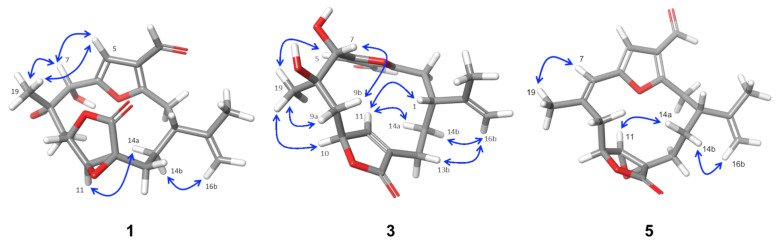
Minimized structures and selected NOE (nuclear Overhauser effect) effects (↔ ) of **1, 3** and **5**.

The relative configurations of compounds **1**−**6** were established by combination of nuclear Overhauser effect spectroscopy (NOESY) experiments, molecular mechanics [26], analysis of key chemical shifts and comparison of their spectroscopic data with those of previously described furanocembranoids [27] (Figure 3).

In compounds **1**, **2** and **6**, the observed NOEs of H-5 with H_3_-19 as well as those of H-7 with H_3_-19 suggested that the vicinal hydroxyl groups at C-7−C-8 should be in a *cis* relationship in both compounds. In compounds **1**, **2** and **6**, the ^1^H NMR signal of H-11 is a singlet due to the roughly 90° dihedral angle formed between H-10 and H-11. Consequently, a small *J*_H-10, H-11_ confirms the relative configuration of C-10 and C-11, which is represented in the energetically favourable conformation shown in Figure 3. The disposition of the isoprenyl groups in both compounds was established from the observed NOEs between H-11 and H-14a and between H-16b and H-14b, situating them on the alpha side of the molecule in compounds **1**, **2** and **6**, as shown in the 3D model (Figure 3 and Figure S13).

In compounds **3** and **4**, the observed NOEs of H_3_-19 with H-5, H-10 and H-11 indicate that these protons and Me-19 must be on same side of the molecule, whereas the NOEs of H_3_-19 with H-9a and of H-7 with H-9b indicate that H-7 and Me-19 have a *trans-*relationship, like the vicinal diols on C-7−C-8. Therefore, the relative configuration at C-8 is opposite to that on compounds **1** and **2**.

In compound **3,** the observed NOEs between H-11 and H-14a; between H-14b and H-16b; and between H-11, H-1 and H-13a and the NOE between H-13b and H-16b in compound **4** all confirmed that the isopropenyl group is situated on the alpha side of the molecule in compounds **3** and **4**.

The configurations of C-7−C-8 vicinal diols were corroborated by comparing the ^1^H and ^13^C NMR chemical shifts around the diol moiety C-7−C-8 in compounds **1** and **3** with those of the related diol compounds [27]. The chemical shifts of C-19 and H-7 in compounds **1** and **3** present strong differences (Δδ_C-19_ = 2.8 ppm and Δδ_H-7_ = 0.69) that are in good agreement with the proposed configurations of the diols, a *cis*-relationship for compound **1** and *trans*-relationship for compound **3** [27]. As in the diol metabolites, the C-7−C-8 configuration of the acetylated compounds **2**, **4** and **6** was also corroborated by comparison of the ^1^H and ^13^C NMR chemical shifts of H-7, H-9, H_3_-19 C-7, C-9 and C-19 (Table 4). The chemical shifts of C-19 and H-7 of compound **4** present strong upfield differences (Δδ_C-19_ ≈ 2.5 ppm and Δδ_H-7_ ≈ 0.6) with respect to **2** and **6**.

In compound **5**, the ^1^H NMR signal for H-11 appears as a singlet due to the roughly 90° dihedral angle formed between H-10 and H-11. This angle confirms the relative configuration of C-10 and C-11, as can be observed in the energetically favourable conformation shown in Figure 3. The observed NOEs between H-11 and H-14a and between H-16b and H-14b indicate that the isopropenyl group is situated on the alpha side of the molecule in compound **5**. Furthermore, the NOE between H_3_-19 and H-7 established the *Z* configuration of the C-7−C-8 double bond, which is in agreement with the chemical shift of C-8 (δ_C_ 129.4 ppm) and C-19 (δ_C-_25.2 ppm) and confirms that **5** is the geometric isomer of isopukalide [14].

### 2.3. Absolute Configuration

The absolute configuration of compound **1** was established by chemical derivatization with (*R*)- and (*S*)-α-methoxyphenyl acetic acids (MPA). NMR analysis of Δδ values obtained for the two MPA esters **1b** and **1c** revealed evidences to assign the absolute configuration at C-7 as *S* (Table 5). Thus, this information established the absolute configuration of **1** as 1*R*, 7*S*, 8*S*, 10*S*, 11*S* and 12*S*.

Compounds **1**−**4** were isolated from a single extract of *Leptogorgia* sp.; therefore, we assume they belong to the same enantiomeric series. Thus, the absolute configurations were assigned as **2**, 1*R*, 7*S*, 8*S*, 10*S*, 11*S* and 12*S*; **3**, 1*R*, 7*S*, 8*R* and 10*S*; and **4**, 1*R*, 7*S*, 8*R* and 10*S*.

Compound **5** was isolated from the same extract that contained pukalide and (*Z*)-deoxypukalide, for which absolute configuration was established previously by NMR using Pirkle’s reagent [14]. Therefore, based on biogenetic considerations and assuming they belong to the same enantiomeric series, the absolute configuration of **5** is assigned as 1*R*, 10*S*, 11*S* and 12*S*.

### 2.4. Furanocembranolides Modulate LPS-Induced Inflammatory Responses on Microglial Cells

**Figure 4:**
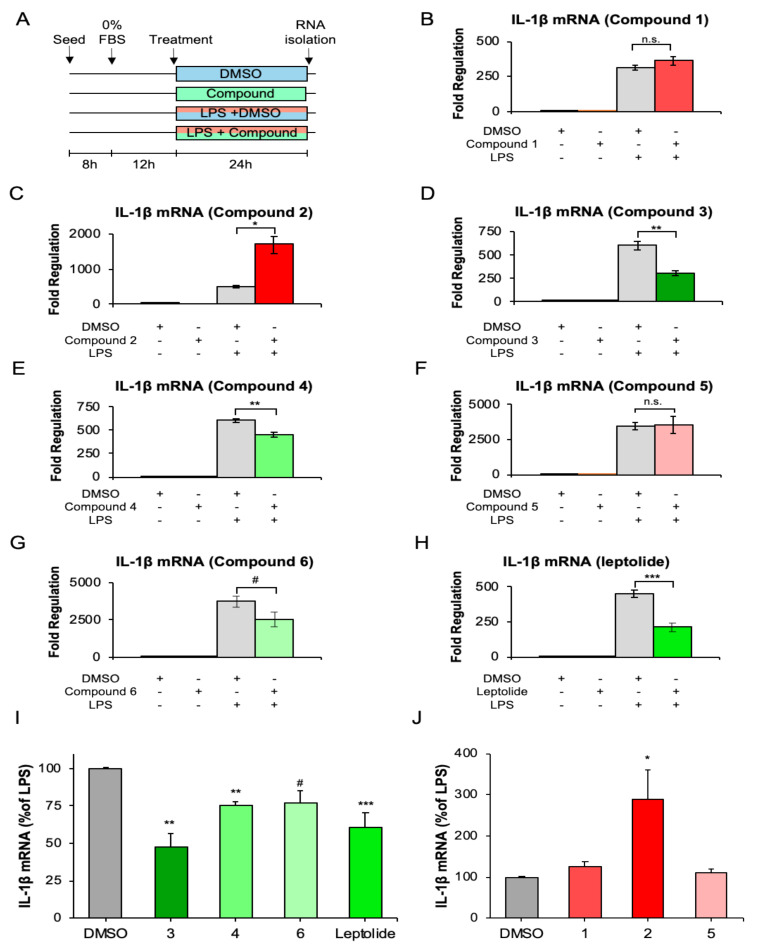
Expression of IL-1β mRNA in vitro after LPS (lipopolysaccharide) stimulation and/or furanocembranolide treatment. (**A**) Experimental design. (**B**–**H**) Representative experiments showing the total fold change of IL-1β mRNA expression in comparison with basal conditions (vehicle alone): Each bar depicts the mean value between quintuplicate qPCR (quantitative real-time polymerase chain reaction) runs, and error bars represent standard deviation. (**I**,**J**) IL-1β mRNA fold regulation normalized to the LPS + DMSO (dimethylsulfoxide) response (considered 100%), separated into furanocembranolides that increase (**I**) or decrease (**J**) the response. Each bar represents the mean between three independent experiments, and error bars show standard error of the mean. Statistical differences were assessed by two-way ANOVA, followed by Holm–Sidak post hoc tests (* *p* < 0.05, ** *p* < 0.01 and *** *p* < 0.001) and by pairwise comparison of raw data by t-test (# *p* = 0.051).

Microglial cells were treated with the carrier (dimethylsulfoxide (DMSO)), the furanocembranolide alone, or with LPS simultaneously with either the carrier or the furanocembranolide (Figure 4A). First, we assessed the effects of these treatments on microglial viability by using the MTT (3-(4,5-dimethylthiazol-2-yl)-2,5-diphenyltetrazolium bromide) colorimetric assay (Figure S14A).

**Figure 5:**
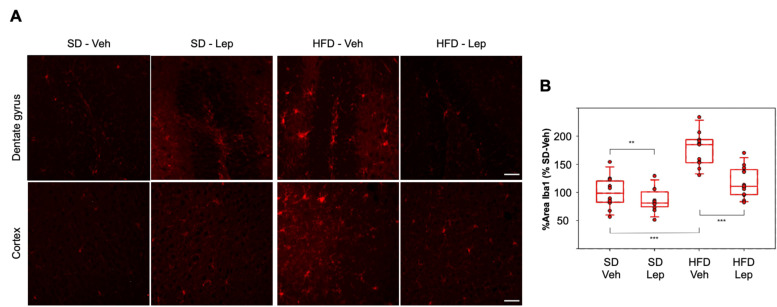
Leptolide treatment ameliorates microgliosis triggered by HFD (high-fat diet): (**A**) Representative immunofluorescence images from paraffin sections of mouse brains stained with Iba1. Calibration bars: 50 μm. (**B**) Immunohistochemical quantification of Iba1 protein expression, measured as percentage of Iba1-positive area per section, normalized to SD-Veh and considered 100% (*n* = 11–13 mice per group): Each dot represents the mean value of Iba1 (ionized calcium-binding adapter molecule 1)-positive area (between dentate gyrus and cortex) for an individual mouse. Statistical differences were assessed by three-way ANOVA followed by Holm–Sidak post hoc tests. * *p* < 0.05, ** *p* < 0.01 and *** *p* < 0.001. Only biologically relevant differences are shown. Abbreviations: SD = Standard Diet; HFD = High-Fat Diet; Veh = Vehicle; Lep = Leptolide.

Among all the furanocembranolides tested in this study, only leptolide significantly increased microglial viability (around 28%). LPS treatments significantly decreased BV2 viability, as expected, with no differences among treatments using compounds or carrier alone. Then, we examined whether treatment with furanocembranolides could reduce LPS-stimulated IL-1β mRNA expression in BV2 cells. LPS stimulation produced a marked increase in IL-1β expression, as expected. In contrast, simultaneous treatment with compound **3** (Figure 4D), **4** (Figure 4E) and leptolide (Figure 4H) caused a marked decrease in IL-1β mRNA synthesis, while compound **6** (Figure 4G) showed a tendency (*p* = 0.051) to diminish inflammation. Compounds **1** (Figure 4B) and **5** (Figure 4F) had no significant effects, whereas compound **2** had a potent pro-inflammatory effect that exacerbated the LPS-induced inflammatory response (Figure 4C). Therefore, it seems that small changes in the functional groups of the furanocembranolide skeleton may have a dramatic effect on the pro- or anti-inflammatory activity. Curiously, three of the compounds induced slight but statistically significant changes in IL-1β mRNA fold regulation in control conditions: compound **2** increased and compound **6** and leptolide decreased IL-1β expression (Figure S14B).

### 2.5. Leptolide Treatment in Vivo Ameliorates Microgliosis in a Mouse Model of Obesity and Insulin Resistance Triggered by HFD (High Fat Diet)

Ionized calcium-binding adapter molecule 1 (Iba1) is a cytoplasmatic protein specifically expressed by microglia, and it is upregulated during microglial activation in various brain diseases, being therefore a marker of microgliosis [28]. Here we found that, in comparison with standard diet (SD), high fat diet (HFD) yielded an increase in Iba1 expression and leptolide treatment significantly reduced Iba1 in both dentate gyrus and cortex (Figure 5A). Moreover, from a qualitative point of view, it was observed that in SD-fed mice microglial cells are smaller and have a branched appearance. This morphology was similar to that attributed to the “resting or surveillant state” of microglia in comparison with those of control HFD-fed mice. These cells are larger and show a more ameboid form associated with an activated, more pro-inflammatory state [6]. Interestingly, in HFD-mice treated with leptolide, microglial cells tend to shift morphologically towards a “resting state” (Figure 5A), indicating a reversion to healthier conditions.

For statistical analyses, we performed three-way ANOVA considering diet, treatment and brain region as factors. All factors proved to be statistically significant, but neither drug nor diet showed dependence on region. There was a significant interaction between diet and drug (*p* = 0.005). Within the SD group, leptolide treatment significantly reduced Iba1 staining below basal levels (SD vehicle). Interestingly, leptolide treatment of HFD-fed mice resulted in a significant decrease in microgliosis, showing very similar behaviour in both brain regions analysed (Figure 5B).

### 2.6. Leptolide Treatment in Vivo Also Reduces Astrogliosis

**Figure 6:**
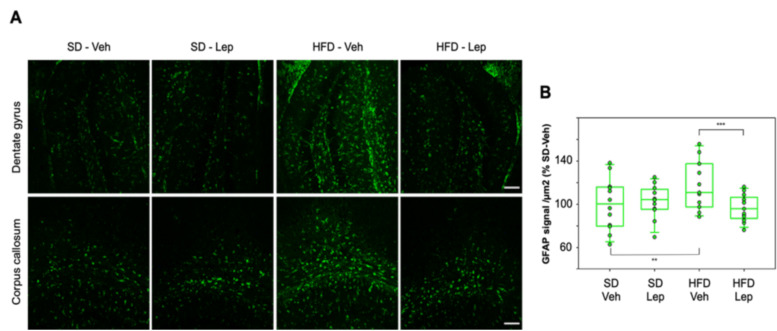
Leptolide treatment ameliorates astrogliosis triggered by HFD: (**A**) Representative immunofluorescence images from paraffin sections of mouse brains stained with GFAP (glial fibrillary acidic protein). Calibration bars: 50μm. (**B**) Immunohistochemical quantification of GFAP expression measured as intensity of fluorescence per area, normalized to SD vehicle, considered as 100% (n = 11–13 mice per group). Each dot represents the mean value of GFAP intensity per area (between dentate gyrus and corpus callosum) for an individual mouse. Statistical differences were assessed by three-way ANOVA followed by Holm–Sidak post hoc tests. * *p* < 0.05, ** *p* < 0.01 and *** *p* < 0.001. Only biologically relevant differences are shown. Abbreviations: SD = Standard Diet; HFD = High Fat Diet; Veh = Vehicle; Lep = Leptolide.

Glial fibrillary acidic protein (GFAP) is an intermediate filament protein expressed by astrocytes and is also considered a good marker of astrogliosis [29]. HFD resulted in an increase in GFAP expression, and this phenotype was partially reverted by treatment with leptolide in both regions studied, with a significant decrease in astrogliosis. Within SD-fed mice, leptolide had no significant effects (Figure 6A). For statistical analyses, we performed three-way ANOVA, as stated before. Diet, drug and region proved to be statistically significant factors. There was a tendency towards interaction between diet and drug (*p* = 0.08). Leptolide treatment of HFD-fed mice resulted in a significant decrease in astrogliosis (Figure 6B).

## 3. Discussion

Many cembranolides have been described to possess anti-inflammatory properties in vitro. For example, durumolides F–K significantly reduced the levels of iNOS protein in an LPS-challenged Raw264.7 macrophagic cell line [30], lobophyolides decreased IL-12 release and attenuated nitric oxide (NO) production in LPS-stimulated dendritic cells [31], and cembrane diterpenoids showed potent anti-inflammatory activity by inhibiting NO production in Raw264.7 cells [32]. However, there are few studies addressing the immune-regulatory properties of cembranolides in glial cells.

In our initial in vitro screening, we evaluated the anti-inflammatory potential of a group of furanocembranolides in LPS-stimulated microglial BV2 cells. Since we were interested in the direct effects of furanocembranolides on microglial early response to a pro-inflammatory challenge, the study focused on transcriptional events. We monitored IL-1β since this cytokine is an “alarmin” or sentinel that detects cellular damage and acts as an amplifier of immune reactions. Thus, IL-1β mRNA constitutes a marker of the inflammasome activation, which controls mature IL-1β production by caspase-1 cleavage. In fact, the rate-limiting step in the processing and secretion of bioactive IL-1β protein takes place with activation of the inflammasome [33]. Interestingly, expression of IL-1β has been demonstrated to be regulated not only at the transcriptional level but also by the stabilization and modulation of the half-life of the generated IL-1β mRNA [34]. In this regard, pro-inflammatory cytokines such as IL-1β itself, IL-6 and TNF-α have been shown to elevate the half-life of IL-1β mRNA [35], while the anti-inflammatory cytokine IL-4 acts as a negative regulator of IL-1β mRNA stability [36]. It has been estimated that, following LPS stimulation, IL-1β mRNA levels rise rapidly, reach their peak at 4 h and then start to decline, but the protein IL-1β can auto-induce itself, maintaining mRNA levels up to 24 h [33]. Our results demonstrate that leptolide and compounds **3**, **4** and **6** can diminish the inflammatory response of LPS-stimulated microglial cells, while compound **2** presented a potent pro-inflammatory effect on microglia. Considering the structures of compounds **1**–**7** and their biological effects on microglia, although they possess the same carbon skeleton, we infer that differences in their functional groups may be critical to reduce or increase LPS-stimulated IL-1β mRNA expression in microglial cells. On comparing leptolide with compound **1**, it seems that substitution of the epoxide at C-7−C-8 with a *cis* diol decreases the anti-inflammatory activity. However, the presence or position of that epoxide is not critical for the activity of class B (CHO) furanocembranolides, since compounds **3** and **4** also exert an anti-inflammatory effect. The observed differences in activity could be related to the configuration at C-8, which in compounds **3** and **4** is the opposite to compounds **1** and **2**. Moreover, comparison of **3** with **4** and of **1** with **2** suggests that substitution with a hydroxyl or an acetate group at C-7 does not substantially affect the potency of these compounds, if the acetate group is in *trans* relationship with the hydroxyl at C-8. However, it has a potent effect if both substituents are in a *cis* relationship. Finally, it is worth noting that compounds **2** and **6** have the same structure, except for the cyano group at C-18, but, while **6** exerts an anti-inflammatory effect, **2** has the opposite activity. This shows the importance of the cyano group at C-18, which is capable of reverting the pro-inflammatory effect of compound **2**.

The in vitro results prompted us to analyse the potential anti-inflammatory effects of these products in vivo. Preclinical models of high-fat diet-induced obesity and insulin resistance display microgliosis and astrogliosis, generating a good model to study central inflammation. We chose leptolide, since we already knew that it has protective effects on systemic insulin resistance in vivo. Furthermore, in this study, leptolide showed a prominent anti-inflammatory activity, along with an increase in microglial viability in vitro. Both microgliosis and astrogliosis, exacerbated by HFD, were diminished by leptolide treatment. In the case of microglia, a morphological shift from a “resting/surveillant state” (SD-mice) to an “activated state” (HFD-vehicle mice) was observed while HFD-fed mice treated with leptolide showed a microglial morphology more similar to the resting state, characteristic of a healthy brain. Moreover, the results regarding gliosis in the dentate gyrus for both glial cell types were also validated in the corpus callosum, the largest white matter structure in the brain, and in the cerebral cortex, which plays an essential role in memory and cognition. We observed the same effects in all regions analysed.

Although gliosis amelioration may be, at least in part, attributed to the decrease in blood levels of inflammatory molecules associated with leptolide treatment [23], our in vitro results support a direct anti-inflammatory effect of leptolide and compounds **3**, **4** and **6** on microglial cells. Interestingly, micheliolide, a sesquiterpene lactone that can suppress LPS-stimulated pro-inflammatory cytokines production in Raw264.7 cells [37], has been demonstrated to cross the blood–brain barrier and to preferentially accumulate in the brain [38]. This finding suggests that leptolide can cross the blood–brain barrier and have both systemic and direct effects on glial cells in vivo.

Leptolide has been shown to act as an insulin sensitizer, enhancing insulin signalling in the liver and muscle of diet-induced obese mice [23]. It is well known that obesity triggers low-grade chronic inflammation, and this compound could partially revert this status, rendering a less inflammatory body environment and a switch towards a lower inflammatory state of the brain. In any case, our results obtained in mice show that leptolide is able to reduce in vivo the gliosis triggered by HFD, which makes leptolide a promising drug to simultaneously treat insulin resistance and neuroinflammation due to its pleiotropic effects on metabolism and gliosis.

To our knowledge, this is the first study to assess the effects of leptolide on gliosis in vivo and of the new furanocembranolides in vitro. Our results on leptolide provide a hopeful therapeutic approach, viable for treating neuroinflammation concomitant with metabolic impairment. However, further research is necessary to address the mechanism by which furanocembranolides exert their therapeutic outcomes on glial cells. Indeed, a better understanding of the mechanisms underlying their anti-neuroinflammatory effects on neurodegenerative processes should lead to more effective treatments in the future.

## 4. Experimental Section

### 4.1. General Experimental Procedures

A Perkin-Elmer model 343 Plus polarimeter (Na lamp at 20 °C) (Perkin-Elmer, Rodgau, Germany) was used for optical rotations measurements. A Perkin-Elmer 1650/FTIR spectrometer (Perkin-Elmer, Rodgau, Germany) was used to record IR spectra. A Bruker AMX 500 instrument (Bruker, Karlsruhe, Germany) operating at 500 MHz for ^1^H NMR and at 125 MHz for ^13^C NMR was used for NMR spectra measurements. The residual solvent signals of deutered chloroform (δ_C_ 77.0 ppm and δ_H_ 7.25 ppm) were used as internal references. The standard Bruker software was used to obtain two-dimensional NMR spectra. A Waters VG-Micromass spectrometer model Zab 2F (Waters, Manchester, UK) was used to obtain the EIMS data. An Agilent 1200 Series Quaternary LC system apparatus was used for high performance liquid chromatography (HPLC) purifications. HPLC detection was carried out by an ultraviolet (UV) detector (DAD G1315D) (Agilent Technologies, Waldbronn, Germany). Two columns were used: an Ascentis^®^ C18 semipreparative column (5 μm, 25 cm × 21.2 mm) which used CH_3_CN-H_2_O mixtures or a Jaigel-sil column (10 μm, 25 cm × 20 mm) which used Hexane-EtOAc mixtures. Stationary phase used for size-exclusion chromatography was Sephadex LH-20, and a mixture of Hexane-MeOH-CH_2_Cl_2_ (3:1:1) was used as solvent system. The mixture H_2_SO_4_-H_2_O-AcOH (1:4:20) was used to visualize thin layer chromatography (TLC) plates.

### 4.2. Collection, Extraction and Isolation

Two collections of *Leptogorgia* specimens were studied in different years. Firstly, from Jicarita (Panama), we collected *Leptogorgia* spp., by Self-Contained Underwater Breathing Apparatus (SCUBA) diving at −15 m. A voucher specimen with code 200511 was deposited at Smithsonian Tropical Research Institute (Panama). A previous report describes the isolation and identification of leptolide together with another five furanocembranolides isolated from *Leptogorgia* spp. [14]. From a later study of the same crude extract, we isolated compound **5** (17.9 mg; t_R_ 32 min) using normal phase HPLC (Hexane-EtOAc (8:2)). Secondly, *Leptogorgia* sp. was similarly collected off Aleta (Panama) at −10 m, depositing another voucher specimen with code 200708 at the Smithsonian (Panama). Specimens (458.3 g) were extracted with acetone at room temperature and concentrated, yielding a dark gum (10.1 g) that was purified by C-18 reversed-phase flash chromatography to obtain fraction 4 (290.4 mg; 2:3 H_2_O/MeOH) that was enriched in cembranolides, as indicated its ^1^H NMR spectrum. This fraction was further chromatographed by Sephadex LH-20 to give four subfractions of interest: 4_a_ (39.4 mg), 4_b_ (27.0 mg), 4_c_ (49.0 mg) and 4_d_ (27.9 mg). Compound **4** (5.2 mg, t_R_ 92 min) was obtained from fraction 4_a_ after C-18 reversed-phase HPLC (H_2_O-CH_3_CN (7:3) → CH_3_CN (100%)). Compound **3** (6.8 mg; t_R_ 51 min) was separated from 4_b_ after C-18 reversed-phase HPLC (H_2_O-CH_3_CN (7:3)). Subfraction 4_c_ (49.0 mg) was chromatographed by C-18 reversed-phase HPLC (H_2_O-CH_3_CN (8:2) → CH_3_CN (100%)) to afford compound **2** (10.2 mg; t_R_ 114 min). Finally, C-18 reversed-phase HPLC (H_2_O-CH_3_CN (9:1) → CH_3_CN (100%)) of subfraction 4_d_ afforded **1** (9.1 mg; t_R_ 105 min).

*Leptogorgodiol A***1**: colourless oil; ${\left[\alpha \right]}_{D}^{20}$ +4.9 (*c* 0.37, CH_2_Cl_2_); IR (film) _max_ 3478, 2936, 1774, 1672 cm^−1^; ^1^H (500 MHz, CDCl_3_) δ 1.34 (3H, *s*, H-19), 1.26 (1H, m, H-14a), 1.39 (1H, m, H-13a), 1.51 (1H, dd, *J* = 11.0, 15.2 Hz, H-9a), 1.77 (3H, *s*, H-17), 1.86 (1H, dd, *J* = 5.0, 15.2 Hz, H-9b), 1.88 (1H, m, H-14b), 2.49 (1H, dd, *J* = 11.2, 14.7 Hz, H-13b), 3.00 (H, m, H-2a), 3.05 (H, m, H-2b), 3.24 (1H, m, H-1), 3.70 (1H, s, H-11), 4.85 (1H, dd, *J* = 5.0, 11.0 Hz, H-10), 4.97 (1H, dd, *J* = 1.6, 1.6 Hz, H-16b), 5.10 (1H, s, H-16b), 5.28 (1H, s, H-7), 6.69 (1H, s, H-5), 9.89 (1H, s, H-18); ^13^C NMR (125 MHz CDCl_3_) δ 18.8 (CH_3_, C-17), 22.5 (CH_3_, C-19), 22.6 (CH_2_, C-13), 29.2 (CH_2_, C-14), 31.9 (CH_2_, C-2), 40.1 (CH, C-1), 40.5 (CH_2_, C-9), 61.0 (C, C-12), 63.0 (CH, C-11), 73.2 (CH, C-7), 74.5 (C, C-8), 74.5 (CH, C-10), 106.4 (CH, C-5), 114.1 (CH_2_, C-16), 123.2 (C, C-4), 144.9 (C, C-15), 155.2 (C, C-6), 162.4 (C, C-3), 172.5 (C, C-20), 184.7 (CH, C-18); EIMS *m/z* 376 [M]^+^, 358 [M-H_2_O]^+^; HREIMS *m/z* [M]^+^ 376.1514 (calcd. for C_20_H_24_O_7_ 376.1522), 358.1412 (calcd. for C_20_H_22_O_6_, 358.1416).

*7-Acetoxy-leptogorgodiol A***2**: colourless oil; ${\left[\alpha \right]}_{D}^{20}$ −11.0 (*c* 0.03, CH_2_Cl_2_); IR (film) _max_ 3473, 2941, 1779, 1678 cm^−1^; ^1^H (500 MHz, CDCl_3_) δ 1.19 (1H, m, H-14a), 1.36 (1H, m, H-13a), 1.39 (3H, s, H-19), 1.45 (1H, dd, *J* = 12.2, 14.9 Hz, H-9a), 1.76 (3H, s, H-17), 1.83 (1H, m, H-14b), 1.86 (1H, dd, *J* = 5.4, 14.9 Hz, H-9b), 2.12 (3H, s, MeCO), 2.47 (1H, dd, *J* = 11.5, 14.7 Hz, H-13b), 3.27 (1H, dd, *J* = 11.2, 11.2 Hz, H-1), 2.96 (1H, dd*, J =* 2.9, 16.8 Hz, H-2a), 3.05 (1H, dd, *J* = 11.8, 16.8 Hz, H-2b), 3.72 (1H, s, H-11), 4.81 (1H, dd, *J* = 5.8, 10.7 Hz, H-10), 4.97 (1H, dd, *J* = 1.6, 1.6 Hz, H-16a), 5.10 (1H, s, H-16b), 6.21 (1H, s, H-7), 6.63 (1H, s, H-5), 9.87 (1H, s, H-18)**;** ^13^C NMR (125 MHz, CDCl_3_) δ 18.8 (CH_3_, C-17), 21.0 (CH_3_, MeCO), 22.5 (CH_2_, C-13), 23.4 (CH_3_, C-19), 28.8 (CH_2_, C-14), 31.6 (CH_2_, C-2), 40.2 (CH, C-1), 40.8 (CH_2_, C-9), 61.0 (C, C-12), 62.9 (CH, C-11), 73.9 (C, C-8), 74.6 (CH, C-7), 74.3 (CH, C-10), 107.0 (CH, C-5), 114.2 (CH_2_, C-16), 124.3 (C, C-4), 144.7 (C, C-15), 151.8 (C, C-6), 162.6 (C, C-3), 170.1 (c, CO), 171.4 (C, C-20), 184.6 (CH, C-18); EIMS *m/z* 418 [M]^+^, 359 [M-CH_3_COO]^+^; HREIMS *m/z* [M]^+^ 418.1619 (calcd. for C_22_H_26_O_8_, 418.1628), 359.1510 (calcd. for C_20_H_23_O_6_, 3359.1495).

*Leptogorgodiol B***3**: colourless oil; ${\left[\alpha \right]}_{D}^{20}$ −4.5 (*c* 0.0.4, CH_2_Cl_2_); IR (film) _max_ 3443, 2940, 1746, 1674 cm^−1^; ^1^H (500 MHz, CDCl_3_) δ 1.40 (3H, s, H-19), 1.77 (3H, s, H-17), 1.49 (1H, m, H-14a), 1.82 (1H, m, H-14b), 1.87 (1H, dd, *J* = 11.6, 14.8 Hz, H-9a), 2.10 (1H, m, H-13a), 2.22 (1H, m, H-1), 2.31 (1H, m, H-13b), 2.57 (1H, dd, *J* = 4.4, 14.8 Hz, H-9b), 2.81 (1H, dd, *J* = 2.2, 14.1, H-2a), 3.09 (1H, dd, *J* = 11.6, 18.4 Hz, H-2b), 4.57 (1H, s, H-7), 4.82 (1H, s, H-16a), 4.87 (1H, dd, *J* = 1.6, 1.6 Hz, H-16b), 4.96 (1H, m, H-10), 5.81 (1H, *s*, H-11), 6.78 (1H, *s*, H-5), 9.94 (1H, *s*, H-18); ^13^C NMR (125 MHz, CDCl_3_) δ 19.2 (CH_3_, C-17), 19.7 (CH_3_, C-19), 21.7 (CH_2_, C-13), 28.5 (CH_2_, C-14), 31.8 (CH_2_, C-2), 43.0 (CH_2_, C-9), 43.8 (CH, C-1), 73.7 (C, C-8), 75.6 (CH, C-7), 78.8 (CH, C-10), 106.4 (CH, C-5), 113.2 (CH_2_, C-16), 125.6 (C, C-4), 133.6 (C, C-12), 145.7 (c, C-15), 148.7 (CH, C-11), 154.6 (C, C-6), 162.9 (C, C-3), 173.5 (C, C-20), 184.6 (CH, C-18); EIMS *m/z* 360 [M]^+^, 342 [M-H_2_O]^+^; HREIMS *m/z* [M]^+^ 360.1561 (calcd. for C_20_H_24_O_6_, 360.1573), [M-CH_3_]^+^ 342.1470 (calcd. for C_20_H_22_O_5_, 342.1467).

*7-Acetoxy-leptogorgodiol B***4**: colourless oil; ${\left[\alpha \right]}_{D}^{20}$ −18.2 (*c* 0.45, CH_2_Cl_2_); IR (film) _max_ 3441, 2915, 1749, 1677 cm^−1^; ^1^H (500 MHz, CDCl_3_) δ 1.48 (3H, s, H-19), 1.49 (1H, m, H-14a), 1.78 (3H, s, H-17), 1.87 (1H, m, H-14b), 1.95 (1H, dd, *J* = 11.9, 15.1 Hz, H-9a), 2.13 (1H, m, H-13a), 2.16 (3H, s, MeCO), 2.25 (1H, m, H-1), 2.36 (1H, m, H-13b), 2.60 (1H, dd, *J* = 4.0, 15.1 Hz, H-9b), 2.89 (1H, dd, *J* = 2.9, 14.8 Hz, H-2a), 3.06 (1H, dd, *J* = 12.2, 14.8 Hz, H-2b), 4.84 (1H, s, H-16a), 4.89 (1H, dd, *J* = 1.6, 1.6 Hz, H-16b), 4.96 (1H, m, H-10), 5.59 (1H, s, H-7), 5.85 (1H, s, H-11),6.70 (1H, s, H-5), 9.95 (1H, s, H-18); ^13^C NMR (125 MHz, CDCl_3_) δ 19.3 (CH_3_, C-17), 21.0 (CH_3_, C-19), 21.1 (CH_3_, MeCO), 21.8 (CH_2_, C-13), 28.7 (CH_2_, C-14), 31.8 (CH_2_, C-2), 43.2 (CH_2_, C-9), 43.8 (CH, C-1), 72.8 (C, C-8), 75.9 (CH, C-7), 78.5 (CH, C-10), 106.9 (CH, C-5), 113.3 (CH_2_, C-16), 125.3 (C, C-4), 133.8 (C, C-12), 145.5 (C, C-15), 148.4 (CH, C-11), 151.5 (C, C-6), 163.5 (C, C-3), 169.5 (C, CO), 171.9 (C, C-20), 184.3 (CH, C-18); EIMS *m/z* 402 [M]^+^, 342 [M-CH_3_COOH]^+^; HREIMS *m/z* [M]^+^ 402.1688 (calcd. for C_22_H_26_O_7_, 402.1679), 342.1468 (calcd. for C_20_H_22_O_5_, 342.1467).

*Z-Isopukalide***5**: colourless oil; ${\left[\alpha \right]}_{D}^{20}$ +66.0 (*c* 0.05, CH_2_Cl_2_); IR (film) _max_ 3441, 2915, 1749, 1677 cm^−1^; ^1^H (500 MHz, CDCl_3_) δ 1.05 (1H, m, H-14a), 1.65 (1H, m, H-14b), 1.59 (1H, m, H-13a), 1.76 (3H, s, H-17), 2.01 (3H, s, H-19), 2.56 (1H, m, H-1), 2.58 (1H, m, H-9a), 2.62 (1H, m, H-13b), 2.83 (1H, dd, *J* = 2.6, 16.6, H-2a), 3.39 (1H, dd, *J* = 12.5, 16.6, H-2b), 3.56 (1H, m, H-9b), 3.81 (1H, s, H-11), 3.82 (3H, s, MeO), 4.60 (1H, dd, *J* = 4.5, 12.8 Hz, H-10), 4.89 (1H, s, H-16a), 4.99 (1H, dd, *J* = 1.3, 1.3 Hz, H-16b), 6.18 (1H, s, H-7), 6.45 (1H, s, H-5); ^13^C NMR (125 MHz, CDCl_3_) δ 19.4 (CH_3_, C-17), 21.7 (CH_2_, C-13), 25.2 (CH_3_, C-19), 28.8 (CH_2_, C-14), 32.8 (CH_2_, C-2), 36.6 (CH_2_, C-9), 43.5 (CH, C-1), 51.7 (CH_3_, OMe), 60.8 (C, C-12), 61.3 (CH, C-11), 76.9 (CH, C-10), 110.4 (CH, C-5), 113.8 (CH_2_, C-16), 115.9 (C, C-4), 117.4 (CH, C-7), 129.4 (C, C-8), 144.3 (C, C-15), 150.4 (C, C-6), 159.8 (C, C-3), 164.3 (CH, C-18), 172.2 (C, C-20); EIMS *m/z* 372 [M]^+^, 340 [M-CH_3_OH]^+^; HREIMS *m/z* [M]^+^ 372.1575 (calcd. for C_21_H_24_O_6_, 372.1573), 340.1310 (calcd. for C_20_H_20_O_5_, 340.1311).

Preparation of compound **6**: leptolide (14.6 mg, 0.045 mmol) was dissolved in 0.5 mL of anhydrous methanol, and while stirring, acetic acid (6.7 mg, 6.5 mL, 0.11 mmol) and NaCN (11 mg, 0.22 mmol) were added. MnO_2_ (78 mg, 0.90 mmol) was added after 1 h of stirring at room temperature. After 21 h, the reaction was filtered through Celite, washed with EtOAc and H_2_O, dried and concentrated. Flash chromatography on silica of the residue obtained allowed to obtain compound **6** (3.6 mg) and compound **2** (2.8 mg).

*Compound***6**: colourless oil; ^1^H (500 MHz, CDCl_3_) δ 1.23 (1H, m, H-14a), 1.38 (1H, m, H-13), 1.40 (3H, s, H-19), 1.55 (1H, m, H-9), 1.74 (3H, s, H-17), 1.81 (1H, m, H-14b), 1.88 (1H, m, H-9), 2.46 (1H, m, H-13), 2.65 (1H, H-2b), 2.73 (1H, m, H-2a), 3.13 (1H, m, H-1), 3.76 (1H, s, H-11), 4.79 (1H, m H-10), 4.95 (1H, s, H-16a), 5.06 (1H, s, H-16b), 5.38/5.39 (1H, s, H-18), 6.17 (1H, s, H-7), 6.45/6.49 (1H, s, H-5); ^13^C NMR (125 MHz, CDCl_3_) δ 18.8 (CH_3_, C-17), 21.1 (CH_3_, MeCO), 22.5 (CH_2_, C-13), 23.6/23.5 (CH_3_, C-19), 28.6/28.5 (CH_2_, C-14), 30.7 (CH_2_, C-2), 40.8/40.9 (CH_2_, C-9), 41.0 (CH, C-1), 56.1 (CH, C-18), 61.0/60.9 (C, C-12), 62.9 (CH, C-11), 73.9/74.0 (C, C-8), 74.6/74.5 (CH, C-10), 75.0/74.9 (CH, C-7), 108.8/108.5 (CH, C-5), 113.9 (CH_2_, C-16), 118.4/118.3 (C, C-4), 117.9/117.6 (C, CN), 145.1 (C, C-15), 150.4/150.2 (C, C-6), 152.2/152.0 (C, C-3), 170.4 (C, *C*H_3_CO), 171.8 (C, C-20); EIMS *m/z* 446 [M + 1]^+^, 418 [M-HCN]^+^; ESMS *m/z* [M + H]^+^ 446.1810 (calcd. for C_23_H_28_NO_8_, 446.1815), 418.1637 (calcd. for C_22_H_26_O_8_, 418.1628).

(*R*)-and (S)-α-methoxyphenyl acetic acids (MPA) ester derivatives **1b** and **1c**. Compound **1** (3.0 mg, 8.0 × 10^−3^ mmol) was dissolved in 1.0 mL of CH_2_Cl_2_ and treated with N,N-dicyclohexycarbodiimide (7.1 mg, 3.4 × 10^−2^ mmol), 4-dimethylaminopyridine (3.5 mg, 2.87 × 10^−2^ mmol) and (*R*)-α-methoxy-α-phenylacetic acid (6.6 mg, 4.0 × 10^−2^ mmol). The reaction was stirred at room temperature for 1 h, and then, after silica gel chromatography (Hexane-EtOAc 1:1) of the reaction mixture, the (*R*)-MPA ester derivative **1b** (1.6 mg, 3.0 × 10^−3^ mmol, 37.5% yield) was obtained. The (*S*)-MPA ester derivative **1c** (1.9 mg, 3.6 × 10^−3^ mmol, 45% yield) was obtained following the same experimental process.

### 4.3. Reagents Preparation

The compounds were dissolved in DMSO (Sigma) to make concentrated stock solutions (ranging from 25 to 100 mM, depending on the amount of product available) within their solubility range. The final concentration of furanocembranolides **1**–**7** added to the cells was 0.1 μM in culture media. The final concentration of leptolide injected into the mice was 0.1 mg/kg in saline. Identical dilutions from the carrier, DMSO, were prepared as controls to rule out the possibility that the effects observed in experimental conditions could be due to the presence of DMSO. The maximal final concentration of DMSO added to the cells was 0.0004% *v/v*. The final concentration of DMSO injected was 0.001% with respect to the mouse plasma volume. These concentrations are below the cytotoxicity threshold.

LPS (Lipopolysaccharide from *E. coli* 0111:B4, L2630, Sigma) was dissolved in PBS (phosphate buffered saline) to make a stock solution (LPS 1 mg/mL). It was then added to the cell cultures at a final concentration of 1 μg/mL.

### 4.4. Cell Culture and Treatments

The murine microglial BV2 cell line was obtained from ATCC (American Type Culture Collection, Virginia, USA). Cells were grown at 37 °C in a humidity-saturated atmosphere containing 5% CO_2_. Culture medium (RPMI Medium 1640 1X (Roswell Park Memorial Institute; Gibco), supplemented with heat-inactivated 5% fetal bovine serum (FBS), 2 mM l-glutamine, 100 U/mL penicillin and 100 U/mL streptomycin) was replaced twice a week, and cells were sub-cultured at 50% confluence.

Experimental protocols were performed as follows: BV2 cells were seeded to a final density of 6500 cells/cm^2^ in a 10 cm Petri dish (Nunc, Thermo Scientific). After 8 h, culture medium was removed, cells were rinsed with PBS (Lonza) and culture medium without FBS was added. Cells were serum starved overnight and then treated for 24 h with different stimuli in serum-free medium: (a) DMSO, (b) furanocembranolide (0.1 μM), (c) LPS (1 μg/mL) plus DMSO and (d) LPS (1 μg/mL) plus furanocembranolide (0.1 μM).

### 4.5. MTT Viability Assay

To measure cell viability, the MTT (3-(4,5-dimethylthiazol-2-yl)-2,5-diphenyltetrazolium bromide) colorimetric assay was performed as previously described [39]. Briefly, cells were seeded in a 96-well standard plate (NUNC, Thermo Scientific), with a cell density of 450 cells/mm^2^, in quadruplicates. After receiving their respective treatments, cells were washed with PBS and treated with MTT (62.5 μg/mL) for 3 h in phenol-red-free DMEM (Dulbecco’s Modified Eagle Medium, Lonza) without FBS. After MTT exposure, cells were incubated in an isopropanol-10% Triton X-100 solution to solubilize formazan. Formazan production was measured by spectrophotometry using the SOFTmax Pro microplate reader (Molecular Devices, San Jose, CA, USA). Absorbance was measured at λ = 570 nm after subtracting the λ = 690 nm background. Measurements were normalized with respect to control conditions.

### 4.6. RNA Isolation from Cell Cultures

At the end of the treatments, cells were rinsed with PBS twice and then collected and lysed with QIAzol Lysis Reagent (Qiagen, Venlo, The Netherlands). Cellular mRNA was extracted following the manufacturer’s protocol. Briefly, QIAzol solution containing lysed cells was mixed with 1:5 volume of chloroform (Merck, Darmstadt, Germany) and mixed vigorously. Then, samples were centrifuged for 15 min at 16,400*g* and two phases were formed. RNA was collected from the aqueous phase and precipitated with isopropanol (Merck). RNA pellets were washed with ethanol 70% (Merck), dried and resuspended in diethyl pyrocarbonate (DEPC)-treated water.

### 4.7. Quantitative Real-Time Polymerase Chain Reaction (RT-qPCR)

RNA concentration was measured with a Nanodrop spectrophotometer (NanoDrop^TM^ N-D1000, Thermo Fisher Scientific, Waltham, MA, USA). Samples were treated with DNase I (0.1 U/µl, Fermentas) to prepare DNA-free RNA. Then, 500 ng of total RNA was reverse-transcribed with Prime-Script (Takara Bio Inc., Otsu, Japan) following the manufacturer’s instructions. The resulting cDNA was used as a template for quantitative real-time polymerase chain reaction (RT-qPCR) using SybrGreen (SYBR Premix Ex Taq kit, Takara Bio Inc., Otsu, Japan). The primers used for RT-qPCR are shown in Table 6.

To validate the specificity of the amplification products, melting curves were analysed and qPCR products were loaded into a 2% agarose gel and run at 100 V for 30 min. IL-1β mRNA fold regulation was calculated for each individual experiment by using the relative quantification strategy of the 2^−∆∆CT^ method [40], using normalization to Rpl18 (housekeeping gene) for each condition. To allow comparisons between different experiments, fold regulation values were normalized by assigning the value 100% to the LPS + DMSO response in order to make it easier to visualize the outcome of each individual compound on IL-1β mRNA modulation. Statistically significant differences of gene transcriptional changes were evaluated by two-way ANOVA, followed by Holm–Sidak post hoc tests. The statistical level of significance was set at *p* < 0.05.

Three different mRNA preparations from three independent cell cultures were assessed for each condition.

### 4.8. Animal Procedures

C57/Bl6J male mice were purchased from Charles River Laboratory (Écully, Lyon, France). Male mice were chosen for metabolic phenotyping to avoid the potential variability related to the oestrous cycle. Experimental procedures were approved by the Animal Care and Use Committee of the University of Valladolid (UVa), Valladolid, Spain, in accordance with the European and Spanish Guidelines for the Care and Use of Mammals in Research. Mice were maintained in positive pressure-ventilated racks at 25 °C with 12 h light/dark cycle, fed ad libitum with standard rodent chow diet and allowed free access to filtered and UV-irradiated water.

At 6 weeks of age, mice were randomly divided into two groups: one of them was fed with standard diet (SD) (33% protein; 58% carbohydrate; 9% fat) (#V1535, Ssniff, Soest, Germany), while the other was fed with a 60% kcal high-fat diet (HFD) (20% protein; 20% carbohydrate; 60% fat) (#D12492, Research Diets, New Brunswick, NJ, USA) for 6 weeks. After this time, each experimental group was randomly divided into two subgroups, which were treated with one-daily intraperitoneal injections of leptolide (0.1 mg/kg of body weight) or vehicle (DMSO) for another 4 weeks. All mice were maintained on their corresponding diet during the 4-week treatment, and finally, they were euthanized at 16 weeks of age. After 4 weeks of treatment, leptolide-treated mice showed improved glucose tolerance by IPGTT (intraperitoneal glucose tolerance test) (area under the curve (AUC): leptolide 36.6 ± 0.8 versus vehicle 39.6 ± 0.8; *p* < 0.05) and improved insulin sensitivity measured by the HOMA index (fasting insulin (μUI/mL) × fasting glucose (mM)/22,5) (leptolide 0.7 ± 0.1 versus vehicle 1.2 ± 0.2; *p* < 0.05).

### 4.9. Immunohistochemistry

Mouse brains were quickly removed after sacrifice and divided into two pieces following a midsagittal section. Brains were fixed by immersion in 4% paraformaldehyde overnight at 4 °C. The tissues were washed in PBS and embedded in paraffin following standard procedures. Coronal slices (5 μm) were cut with a rotary microtome (Microm, Wayzata, MN, USA), serially mounted on Polysine™ slides (Menzel-Gläser) and dried. Sample processing was performed by CNB Histology Facility (UAM-CSIC, Madrid, Spain).

Tissue slices were dewaxed in xylene and rehydrated through an ethanol series into phosphate buffered saline (PBS). The slices were then blocked and permeabilized with Triton X-100 (0.25% in PBS) and 1% normal goat serum. The following primary antibodies were used: mouse anti-GFAP (1 μg/mL, Santa Cruz Biotechnology, Dallas, TX, USA) and rabbit anti-Iba1 (0.5 μg/mL, Wako, Germany). As secondary antibodies, Alexa Fluor 488-conjugated goat anti-mouse IgG and Alexa Fluor 594-conjugated goat anti-rabbit IgG (1.5 μg/mL, Jackson Immunoresearch, West Grove, PA, USA) were used.

### 4.10. Image Acquisition

Immunostained sections were visualized with an Eclipse 90i fluorescence microscope (Nikon) equipped with a DS-Ri1 (Nikon, Minato, Tokyo, Japan) digital camera. Images were acquired under the same conditions of illumination, diaphragm and condenser adjustments, exposure time, background correction and colour levels.

Images were acquired from dentate gyrus for both astrocytes and microglia since, as part of the hippocampus, it is considered to play a crucial role in associative memory. To validate the results obtained, an additional region for each cell type was also analysed: corpus callosum for astrocytes and cortex for microglia. For each region, two different series 20 µm apart were examined.

### 4.11. Image Quantification

Astroglial and microglial fluorescence levels were quantified with ImageJ software. For astrocytosis quantification, GFAP immunoreactive intensity has a well-known correlation with astrocyte reactivity [41]; therefore, astrogliosis was measured as a GFAP fluorescence intensity per unit area. For microgliosis measurements, Iba1-positive areas were calculated in each photograph, since microgliosis is correlated with morphological changes that imply an increase in cell volume [42]. The microglial reactivity was measured in terms of percentage of area Iba1-positive per section.

### 4.12. Statistical Analysis

Statistical analyses were performed with SigmaPlot v.11.0 (Systat, San Jose, CA, USA) software. A *p* value < 0.05 was used as a threshold for significant changes. The tests used for each experiment are stated in figure legends.

## Figures and Tables

**Table 1:** ^1^H and ^13^C NMR spectroscopic data (500 and 125 MHz, CDCl_3_) of compounds **1** and **2**.

No.	1		2	
	δ_C_, Type	δ_H_ (*J* in Hz)	δ_C_, Type	δ_H_ (*J* in Hz)
1	40.1, CH	3.24, m	40.2, CH	3.27, dd (11.2, 11.2)
2	31.9, CH_2_	3.00, m 3.05, m	31.6, CH_2_	2.96, dd (2.9, 16.8) 3.05, dd (11.8, 16.8)
3	162.4, C	-	162.6, C	-
4	123.2, C	-	124.3, C	-
5	106.4, CH	6.69, s	107.0, CH	6.63, s
6	155.2, C	-	151.8, C	-
7	73.2, CH	5.28, s	74.6, CH	6.21, s
8	74.5, C	-	73.9, C	-
9	40.5, CH_2_	1.51, dd (11.0, 15.2) 1.86, dd (5.0, 15.2)	40.8, CH_2_	1.45, dd (12.2, 14.9) 1.86, dd (5.4, 14.9)
10	74.5, CH	4.85, dd (5.0, 11.0)	74.3, CH	4.81, dd (5.8, 10.7)
11	63.0, CH	3.70, s	62.9, CH	3.72, s
12	61.0, C	-	61.0, C	-
13	22.6, CH_2_	a: 1.39, m b: 2.49, dd (11.2, 14.7)	22.5 CH_2_	a: 1.36, m b: 2.47, dd (11.5, 14.7)
14	29.2, CH_2_	a: 1.26, m b: 1.88, m	28.8 CH_2_	a: 1.19, m b: 1.83, m
15	144.9, C	-	144.7, C	-
16	114.1, CH_2_	a: 4.97, dd (1.6, 1.6) b: 5.10, s	114.2, CH_2_	a: 4.97, dd, (1.6, 1.6) b: 5.10, s
17	18.8, CH_3_	1.77, s	18.8 CH_3_	1.76, s
18	184.7, CH	9.89, s	184.6, CH	9.87, s
19	22.5, CH_3_	1.34, s	23.4, CH_3_	1.39, s
20	172.5, C	-	171.4, C	-
7-CH_3_*C*O			170.1, C	-
7-*CH_3_*CO			21.0, CH_3_	2.12, s

**Table 2:** ^1^H and ^13^C NMR spectroscopic data (500 and 125 MHz, CDCl_3_) of compounds **3** and **4**.

No.	3		4	
	δ_C_, Type	δ_H_ (*J* in Hz)	δ_C_, Type	δ_H_ (*J* in Hz)
1	43.8, CH	2.22, m	43.8, CH	2.25, m
2	31.8, CH_2_	2.81, dd (2.2, 14.1) 3.09, dd (11.6, 14.8)	31.8, CH_2_	2.89, dd (2.9, 14.8) 3.06, dd (12.2, 14.8)
3	162.9, C	-	163.5, C	-
4	125.6, C	-	125.3, C	-
5	106.4, CH	6.78, s	106.9, CH	6.70, s
6	154.6, C	-	151.5, C	-
7	75.6, CH	4.57, s	75.9, CH	5.59, s
8	73.7, C	-	72.8, C	-
9	43.0, CH	b: 1.87, dd (11.6, 14.8) a: 2.57, dd (4.4, 14.8)	43.2, CH_2_	b: 1.95, dd (11.9, 15.1) a: 2.60, dd (4.0, 15.1)
10	78.8, CH	4.96, m	78.5, CH	4.96, m
11	148.7, CH	5.81, s	148.4, CH	5.85, s
12	133.6, C	-	133.8, C	-
13	21.7 CH_2_	a: 2.10, m b: 2.31, m	21.8, CH_2_	a: 2.13, m b: 2.36, m
14	28.5 CH_2_	a: 1.49, m b: 1.82, m	28.7, CH_2_	a: 1.49, m b: 1.87, m
15	145.7, C	-	145.5, C	-
16	113.2, CH_2_	a: 4.82, s b: 4.87, dd (1.6, 1.6)	113.3, CH_2_	a: 4.84, s b: 4.89, dd (1.6, 1.6)
17	19.2 CH_3_	1.77 s, br s	19.3, CH_3_	1.78, s
18	184.6, CH	9.94, s	184.3, C	9.95, s
19	19.7, CH_3_	1.40, s	21.0, CH_3_	1.48, s
20	173.5, C	-	171.9, C	-
7-CH_3_*C*O			169.5, C	-
7-*CH_3_*CO			21.1, CH_3_	2.16, s

**Table 3:** ^1^H and ^13^C NMR spectroscopic data (500 and 125 MHz, CDCl_3_) of compounds **5**–**6**.

No.	5		6 *	
	δ_C_, Type	δ_H_ (*J* in Hz)	δ_C_, Type	δ_H_ (*J* in Hz)
1	43.5, CH	2.56 m	41.0, CH	3.13, m
2	32.8, CH_2_	2.83, dd (2.6, 16.6) 3.39, dd (12.5, 16.6)	30.7, CH_2_	2.73, m 2.65, m
3	159.8, C	-	152.0 / 152.2, C	-
4	115.9, C	-	118.4 / 118.3, C	-
5	110.4, CH	6.45, s	108.5 / 108.8, CH	6.45 / 6.49, s
6	150.4, C	-	150.2 / 150.4, CH	-
7	117.4, CH	6.18, s	75.0 / 74.9, CH	6.17, s
8	129.4, C	-	73.9 / 74.0, C	-
9	36.6, CH_2_	3.56, m 2.58, m	40.9 / 40.8, CH	1.88 m 1.55, m
10	76.9, CH	4.60, dd (4.5, 12.8)	74.6 / 74.5, CH	4.79, m
11	61.3, CH	3.81, s	62.9, CH	3.76, s
12	60.8, C	-	60.9 / 61.0, C	-
13	21.7, CH_2_	1.59, m 2.62, m	22.5, CH_2_	1.38, m 2.46, m
14	28.8, CH_2_	a: 1.05, m b: 1.65, m	28.5 / 28.6, CH_2_	a: 1.23, m b: 1.81, m
15	144.3, C	-	145.1, C	-
16	113.8, CH_2_	a: 4.89, s b: 4.99, dd (1.6, 1.6)	113.9, CH_2_	a: 4.95, s b: 5.06, s
17	19.4, CH_3_	1.76, s	18.8, CH_3_	1.74, s
18	164.3, C	-	56.1, CH	5.38 / 5.39, s
19	25.2, CH_3_	2.01, s	23.5 / 23.6, CH_3_	1.40, s
20	172.2, C	-	171.8, C	-
21	51.7, CH_3_	3.82 s		-
7-CH_3_*C*O			170.4, C	
7-*CH_3_*CO			21.1, CH_3_	2.15, s
18-*CN*			117.9 / 117.6, C	

* NMR data of the mixture of epimers at C-18.

**Table 4:** Selected ^1^H and ^13^C NMR data (CDCl_3_) of compounds **2** and **4** and epimers **6**.

	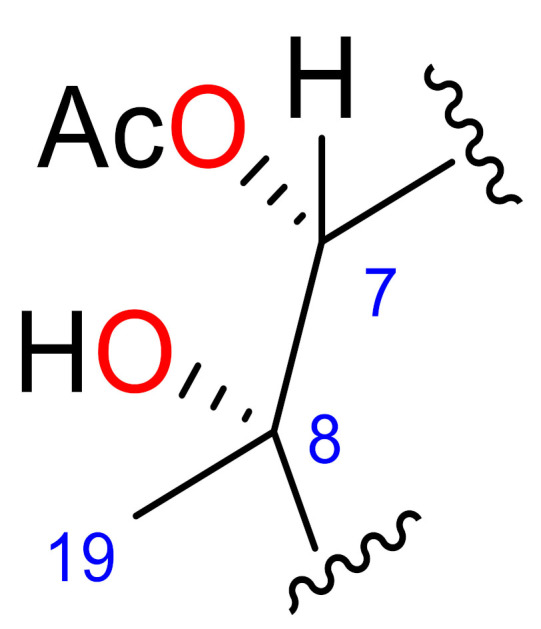		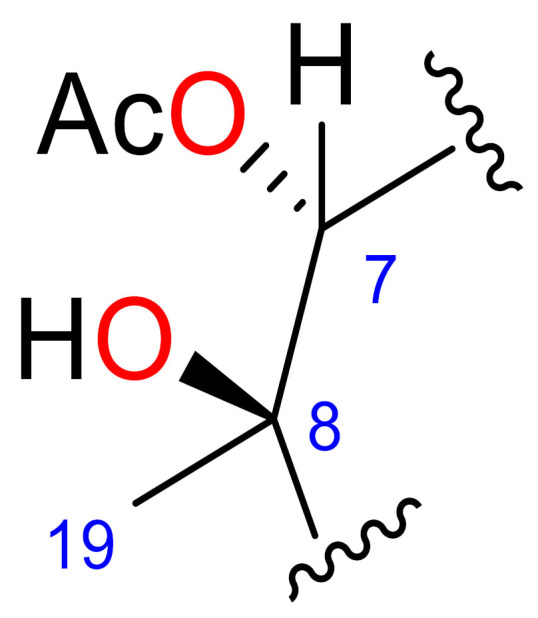
No.	**2**	**6**	**4**
δ_H-7_	6.21, s	6.17, s	5.59, s
δ_H-9_	1.45, dd 1.86, dd	1.55, m 1.88 m	1.95, dd 2.60, dd
δ_H-19_	1.39, s	1.35, s	1.48 s
δ_C-7_	74.6	74.0	75.9
δ_C-9_	40.8	40.5	43.2
δ_C-19_	23.4	22.4	21.0

**Table 5:** ^1^H NMR Δδ (δ_R_−δ_S_) values (CDCl_3_, ppm, recorded at 500 MHz) of the diastereomeric (*S*)-α-methoxyphenyl acetic acids (MPA) esters **1b** and **1c**.

No.	δ_R_	δ_S_	Δδ_R-S_
δ_H-18_	9.84	9.77	+0.070
δ_H-5_	6.53	6.08	+0.45
δ_H-19_	1.10	1.30	−0.20
δ_H-10_	4.60	4.72	−0.12

**Table 6:** Primers used for quantitative real-time polymerase chain reaction (RT-qPCR).

Gene Name (ID)	Primer Name	Sequence (5′-3′)
IL-1β (NM_008361.4)	Mouse IL1β-Forward	TGTAATGAAAGACGGCACACCCAC
	Mouse IL1β-Reverse	GGCTTGTGCTCTGCTTGTGAGG
Rpl18 (NM_009077.2)	Mouse Rpl18-Forward	TTCCGTCTTTCCGGACCT
	Mouse Rpl18-Reverse	TCGGCTCATGAACAACCTCT

## Supplementary Materials

The following are available online at https://www.mdpi.com/1660-3397/18/8/378/s1, Figure S1: ^1^H NMR of **1** in CDCl_3_; Figure S2: ^13^C NMR spectrum of **1** in CDCl_3_; Figure S3: ^1^H NMR of **2** in CDCl_3_; Figure S4: ^13^C NMR spectrum of **2** in CDCl_3_; Figure S5: ^1^H NMR of **3** in CDCl_3_; Figure S6: ^13^C NMR spectrum of **3** in CDCl_3_; Figure S7: ^1^H NMR of **4** in CDCl_3_; Figure S8: ^13^C NMR spectrum of **4** in CDCl_3_; Figure S9: ^1^H NMR of **5** in CDCl_3_; Figure S10: ^13^C NMR spectrum of **5** in CDCl_3_. Figure S10: ^1^H NMR of **6** in CDCl_3_; Figure S11: ^13^C NMR spectrum of **6** in CDCl_3_; Figure S13: Minimized structures and selected NOE effects of **2**, **4** and **6**; Figure S14: Effects of furanocembranolides on microglial cell viability and inflammation.
