# Supplementary material for: Modulation of Glial Responses by Furanocembranolides: Leptolide Diminishes Microglial Inflammation in Vitro and Ameliorates Gliosis In Vivo in a Mouse Model of Obesity and Insulin Resistance
Source: Mar Drugs. 2020 Jul 22;18(8):378. doi: 10.3390/md18080378 (PMC7459604; doi:10.3390/md18080378)
Supplement: Supplementary file 1 [file marinedrugs-18-00378-s001.pdf]

marine drugs

Supplementary Materials

Modulation of glial responses by furanocembranolides: leptolide diminishes microglial inflammation in vitro and ameliorates gliosis in vivo in a mouse model of obesity and insulin resistance

Miriam Corraliza-Gómez [1,†], Amalia B. Gallardo [2,3,†], Ana R. Díaz-Marrero [2,‡], José M. de la Rosa [2,¥], Luis D'Croz [4,5], José Darias [2], Eduardo Arranz [1], Irene Cózar-Castellano [1,6], María D. Ganfornina [1,*] and Mercedes Cueto [2,*]

1 Instituto de Biología y Genética Molecular, Universidad de Valladolid-CSIC, 47003 Valladolid, Spain
2 Instituto de Productos Naturales y Agrobiología (IPNA-CSIC). Avenida Astrofísico F. Sánchez, 3, 38206 La Laguna, Tenerife, Spain
3 Facultad de Ciencias, Universidad de Magallanes, Departamento de Ciencias y Recursos Naturales, Avenida Bulnes 01855, Punta Arenas, Chile
4 Departamento de Biología Marina y Limnología, Universidad de Panamá, Panama
5 Smithsonian Tropical Research Institute, STRI, Box 0843-03092, Balboa, Panama
6 Centro de Investigación Biomédica en Red de Diabetes y Enfermedades Metabólicas Asociadas (CIBERDEM), Madrid, Spain
* Correspondence: opabinia@ibgm.uva.es; Tel.: +34-983-184-814; mcueto@ipna.csic.es; Tel.: +34-922-250-144
† These authors contributed equally to this work
‡ Present address: Instituto Universitario de Bio-Orgánica Antonio González (IUBO AG), Universidad de La Laguna (ULL), Avda. Astrofísico F. Sánchez 2, 38206 La Laguna, Tenerife, Spain¥ Present address: Departmento de Bioquímica, Microbiología, Biología Celular and Genética, Facultad de Farmacia, Universidad de La Laguna (ULL), 38206 La Laguna, Tenerife, Spain

TABLE OF CONTENTS.

**Figure S1. ^{1}H NMR spectrum of 1 in $CDCl_3$**	**S2**
**Figure S2.** ^{13}C NMR spectrum of **1** in $CDCl_3$	**S3**
**Figure S3.** ^{1}H NMR spectrum of **2** in $CDCl_3$	**S4**
**Figure S4.** ^{13}C NMR spectrum of **2** in $CDCl_3$	**S5**
**Figure S5.** ^{1}H NMR spectrum of **3** in $CDCl_3$	**S6**
**Figure S6.** ^{13}C NMR spectrum of **3** in $CDCl_3$	**S7**
**Figure S7.** ^{1}H NMR spectrum of **4** in $CDCl_3$	**S8**
**Figure S8.** ^{13}C NMR spectrum of **4** in $CDCl_3$	**S9**
**Figure S9.** ^{1}H NMR spectrum of **5** in $CDCl_3$	**S10**
**Figure S10.** ^{13}C NMR spectrum of **5** in $CDCl_3$	**S11**
**Figure S11.** ^{1}H NMR spectrum of **6** in $CDCl_3$	**S12**
**Figure S12.** ^{13}C NMR spectrum of **6** in $CDCl_3$	**S13**
**Figure S13.** Minimized structures and selected NOE effects of **2**, **4** and **6**.	**S14**
**Figure S14.** Effects of furanocembranolides on microglial cell viability and inflammation.	**S15**

Figure S1. ^{1}H NMR of leptogorgodiol A **1** in $CDCl_3$

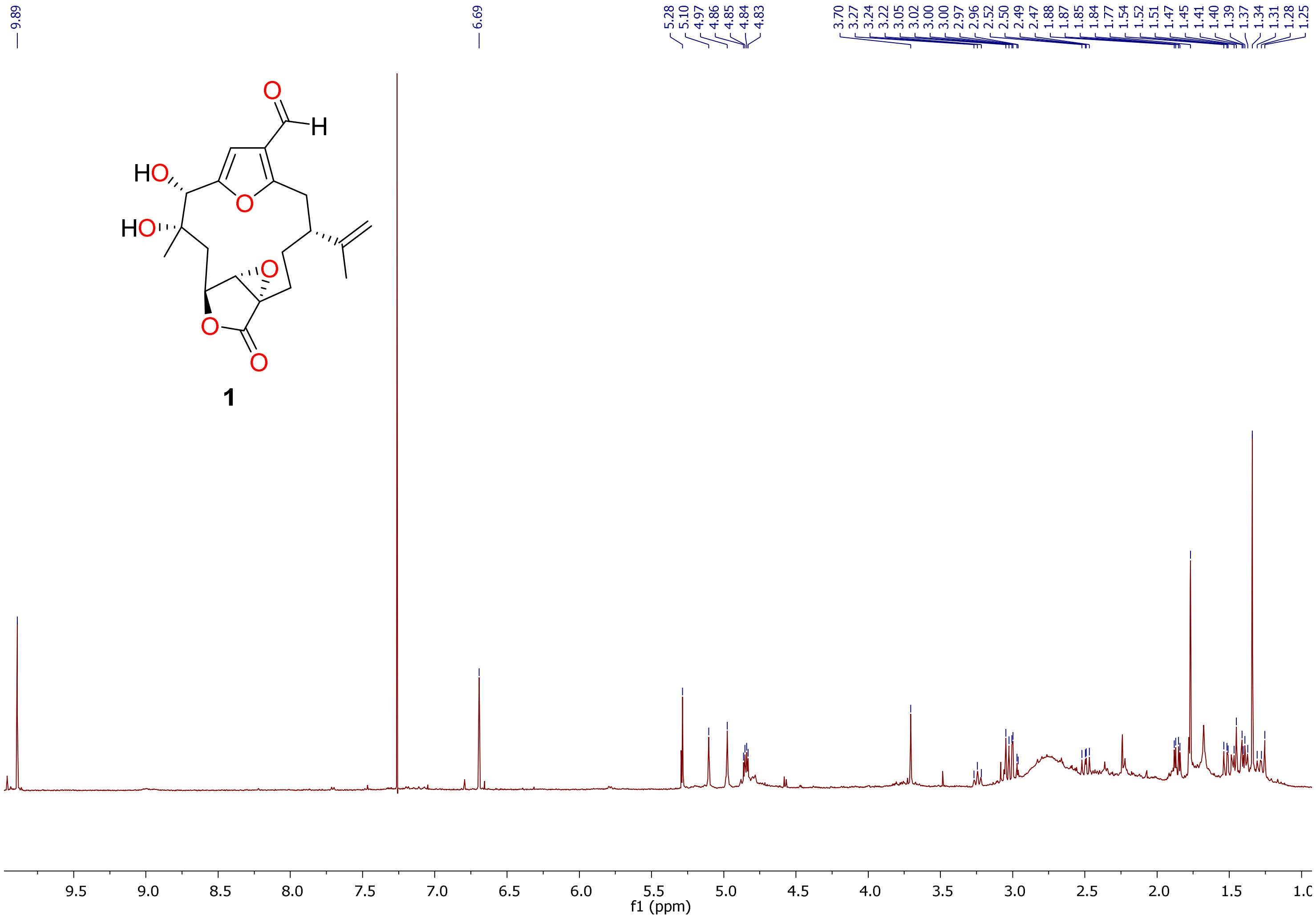

Figure S2. ^{13}C NMR spectrum of leptogorgodiol A **1** in $CDCl_3$

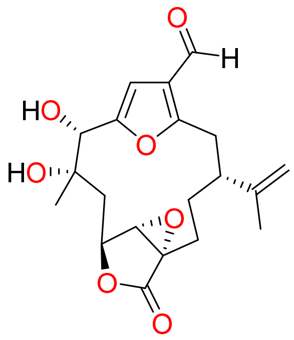

1

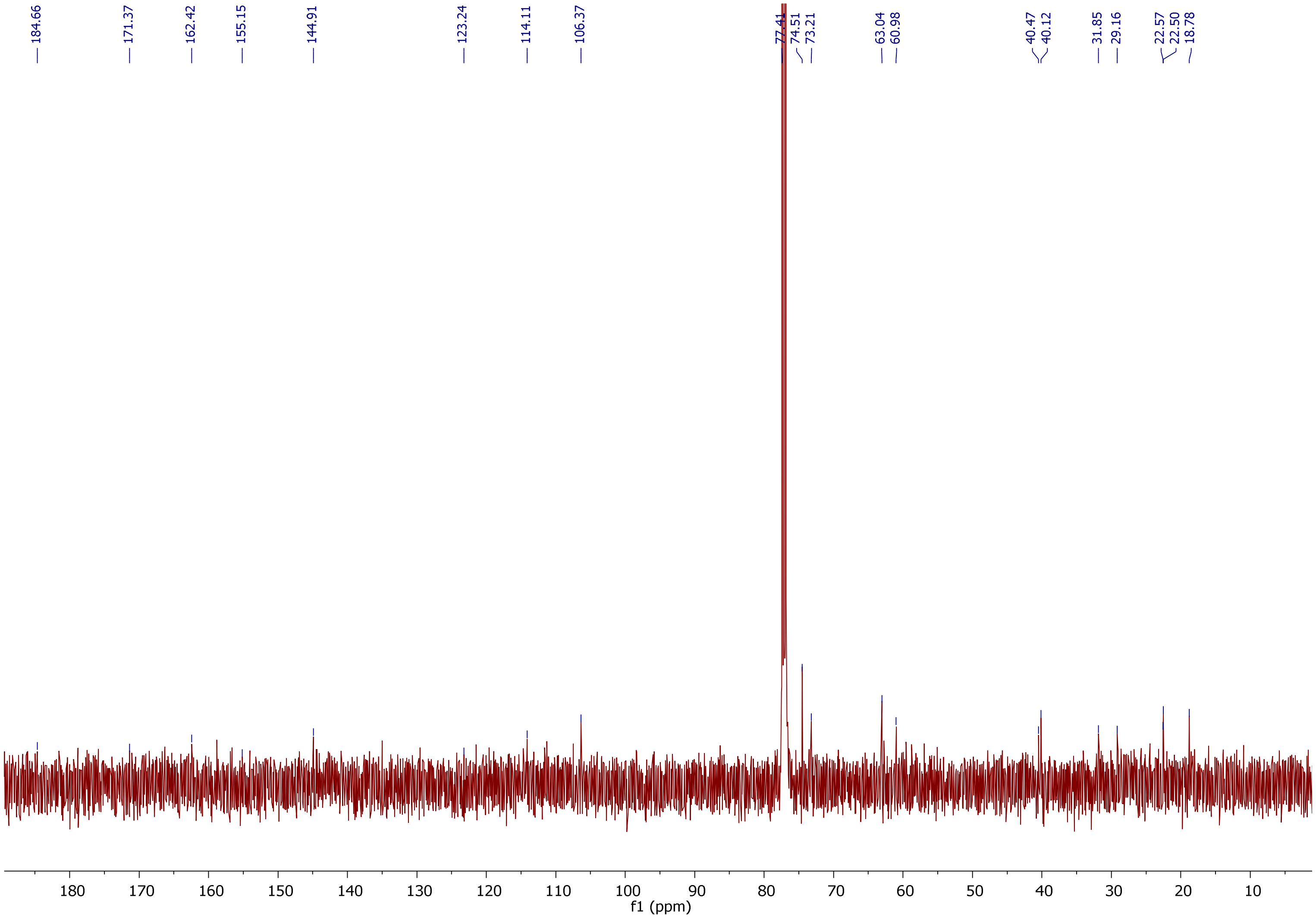

184.66
171.37
162.42
155.15
144.91
123.24
114.11
106.37
77.41
74.51
73.21
63.04
60.98
40.47
40.12
31.85
29.16
22.57
22.50
18.78
180
170
160
150
140
130
120
110
100
90
80
70
60
50
40
30
20
10
f1 (ppm)

Figure S3. ^{1}H NMR of 7-acetate-leptogorgodiol A **2** in $CDCl_3$

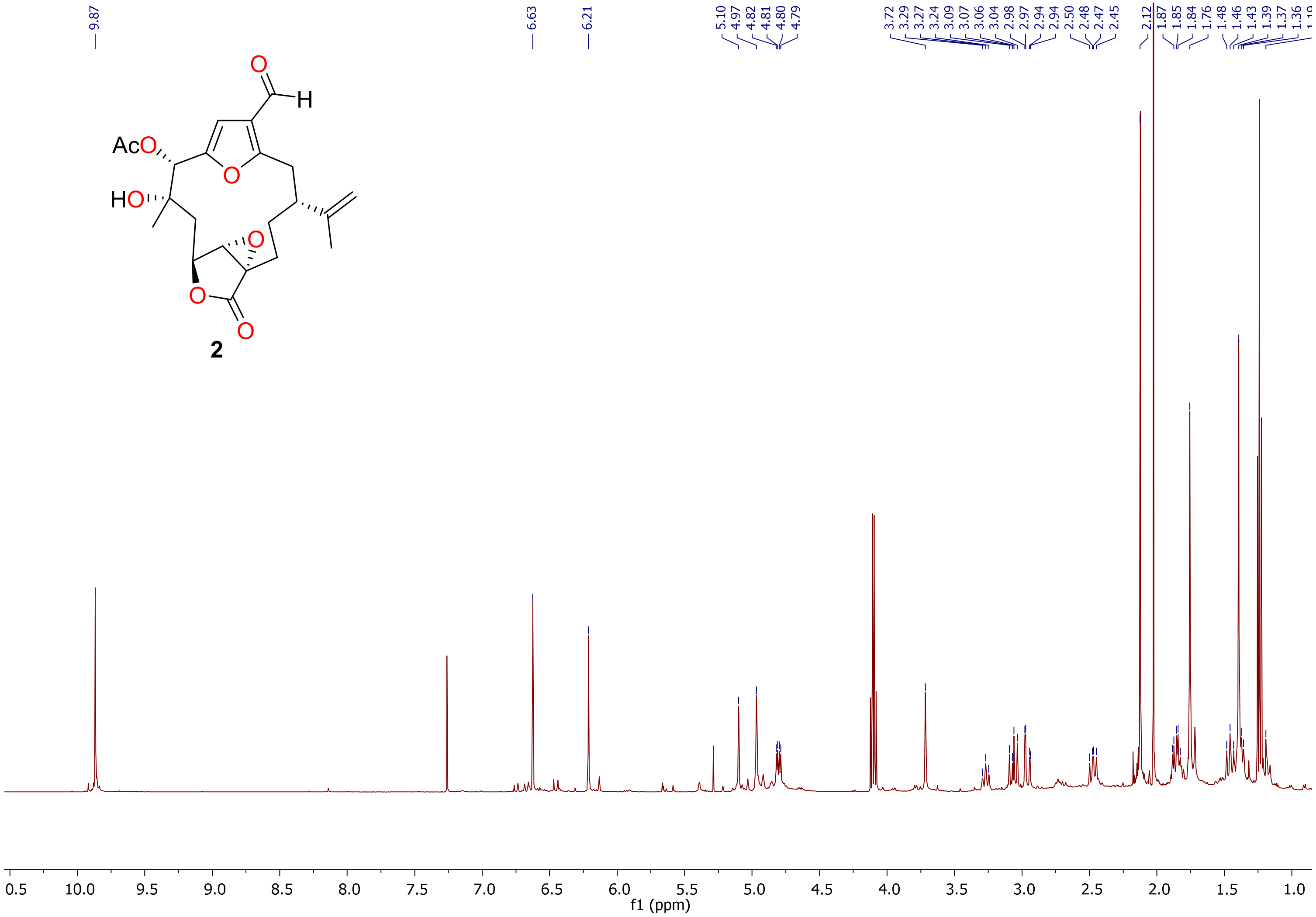

Figure S4. ^{13}C NMR spectrum of 7-acetate-leptogorgodiol A **2** in $CDCl_3$

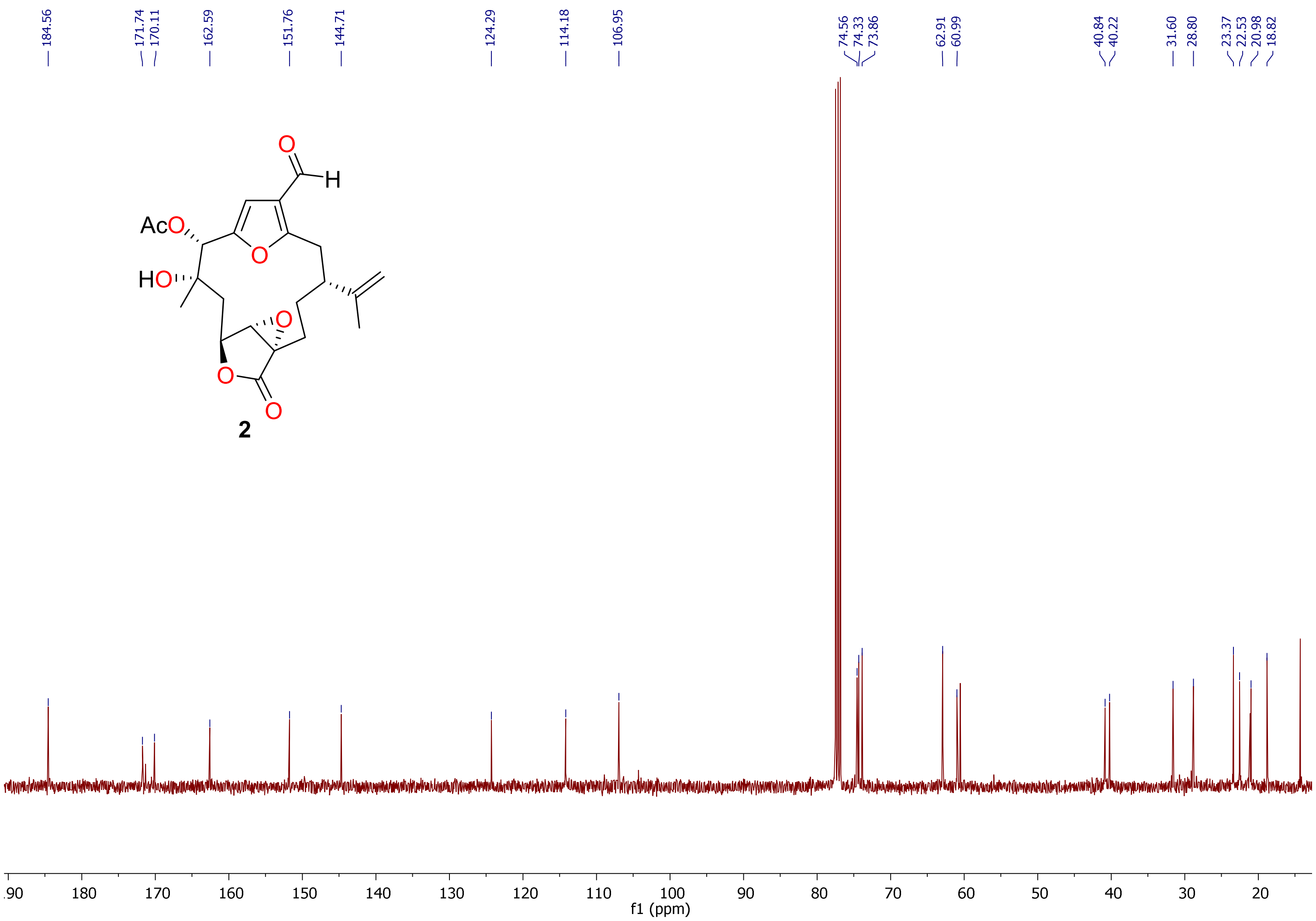

Figure S5. ^{1}H NMR of leptogorgodiol B **3** in $CDCl_3$

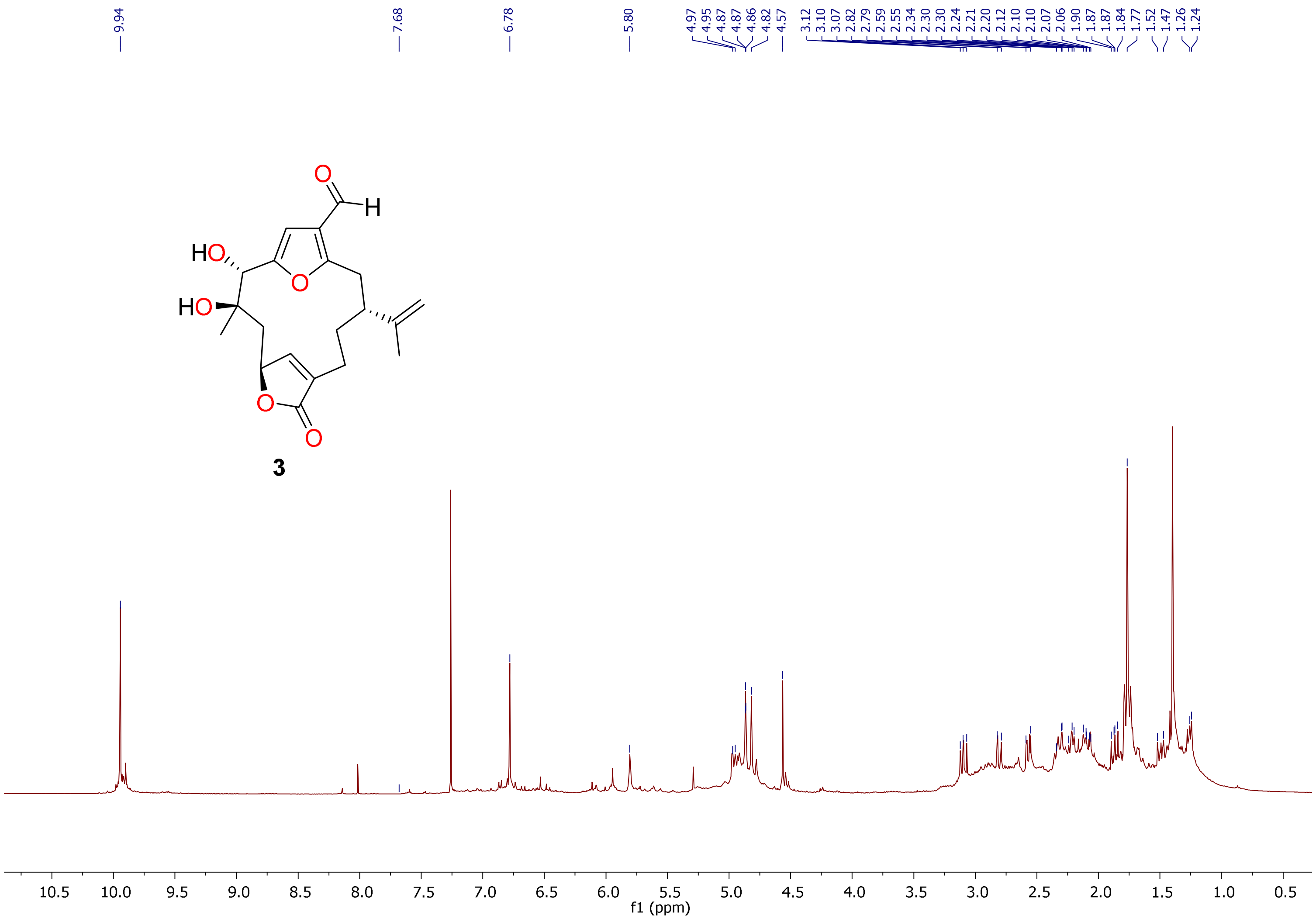

Figure S6. ^{13}C NMR spectrum of leptogorgodiol B **3** in $CDCl_3$

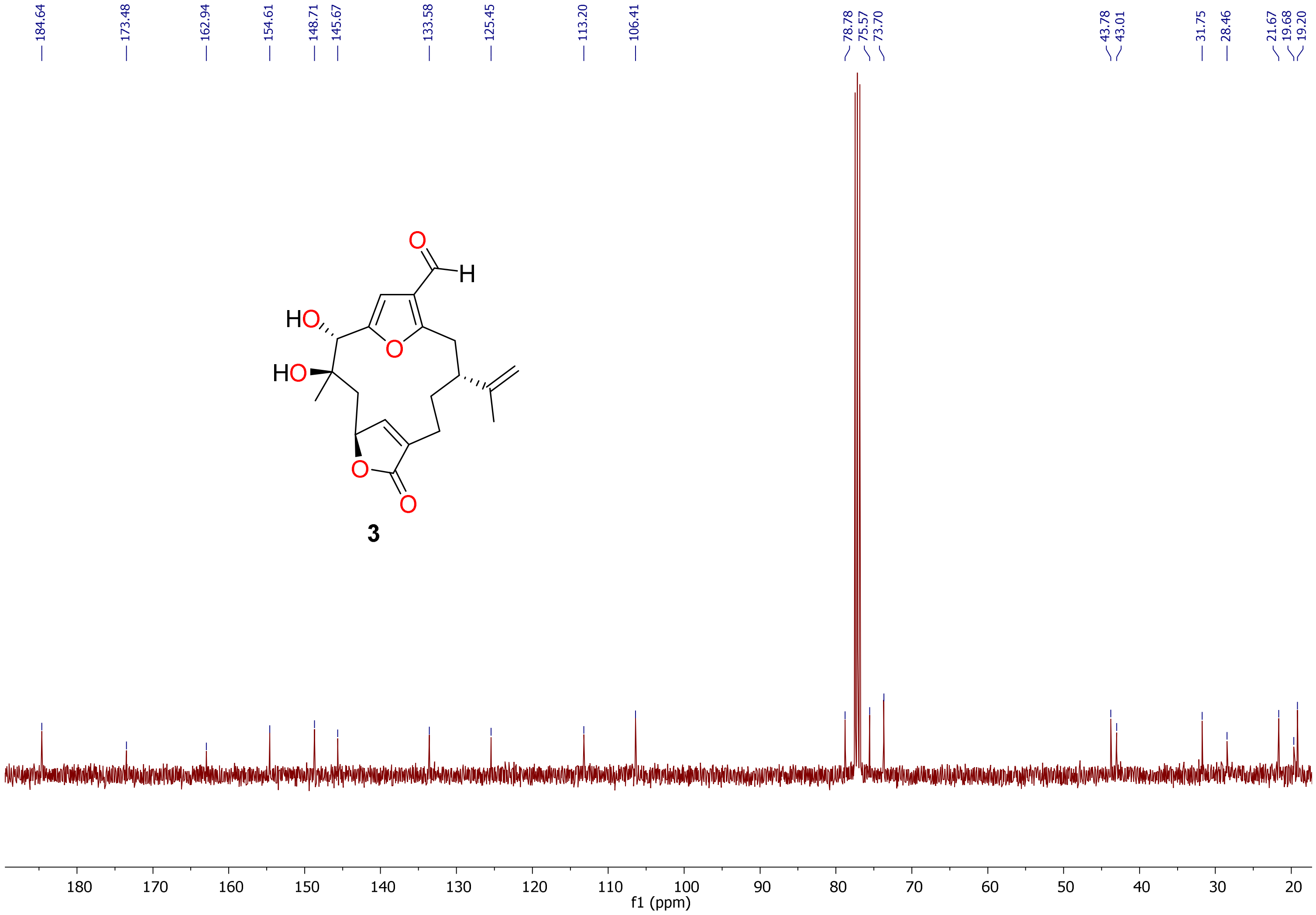

Figure 7. ^{1}H NMR of 7-acetate-leptogorgodiol B **4** in $CDCl_3$.

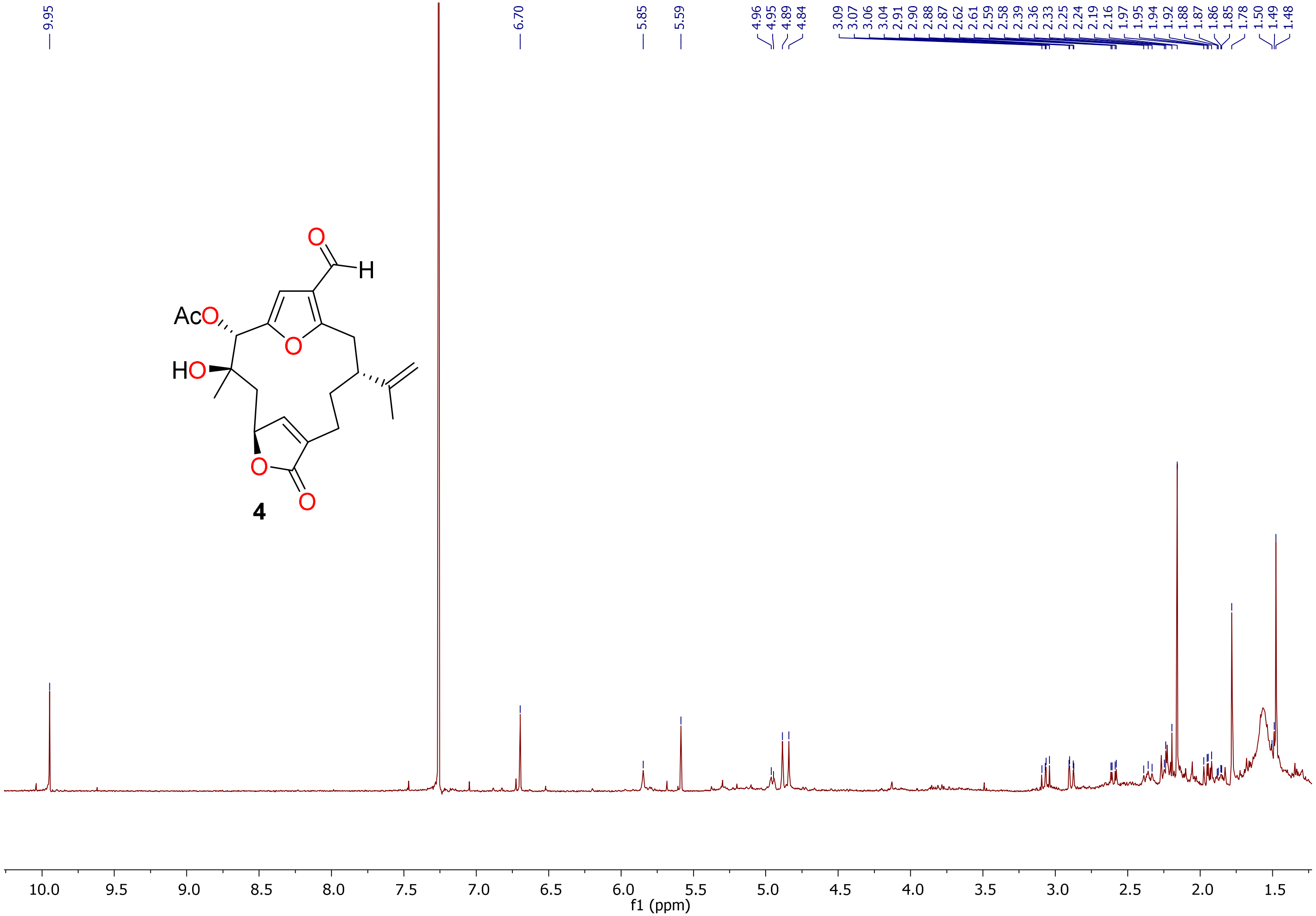

Figure S8. ^{13}C NMR spectrum of 7-acetate-leptogorgodiol B **4** in $CDCl_3$

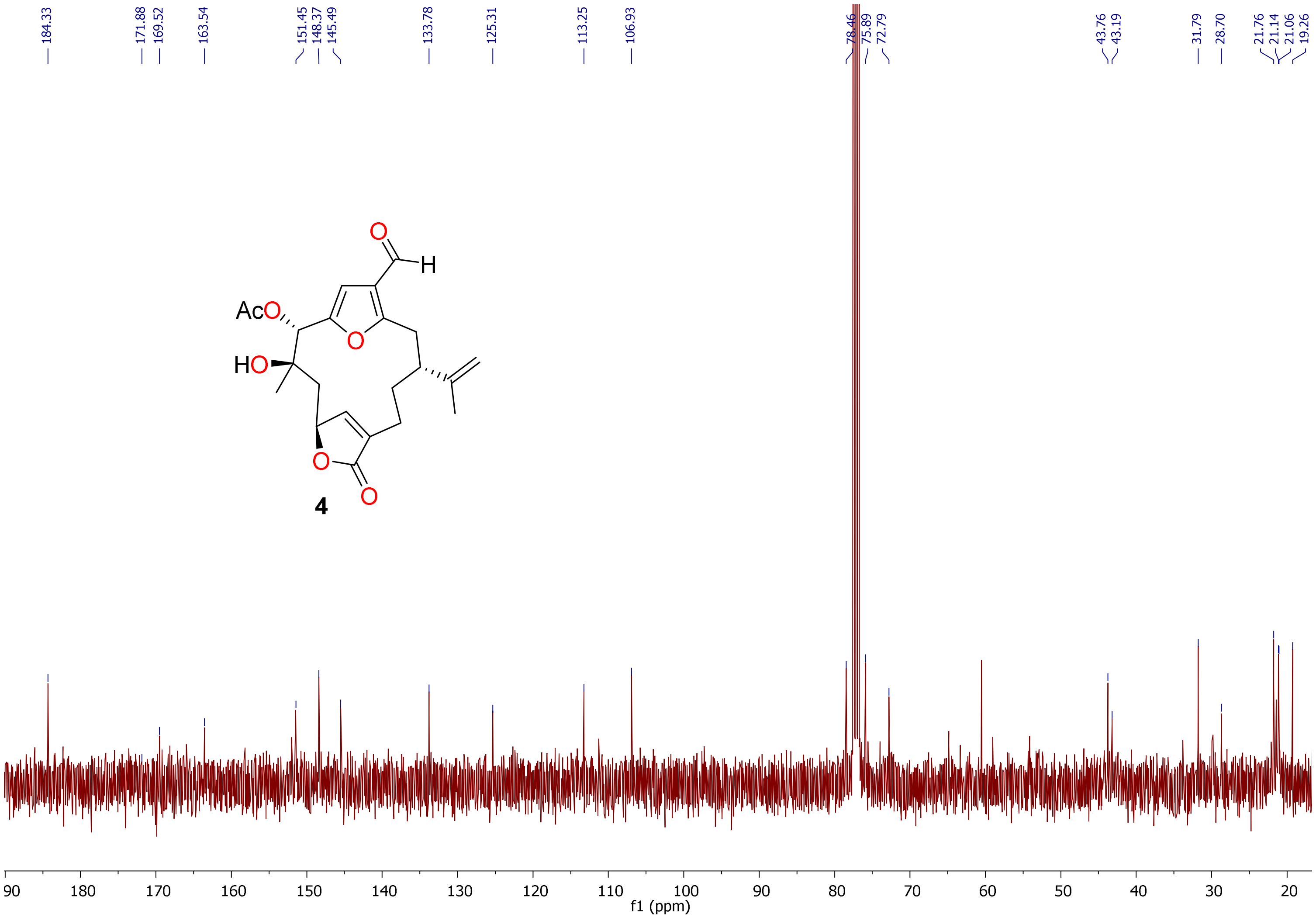

Figure 9. ^{1}H NMR of *Z*-isopukalide **5** in $CDCl_3$.

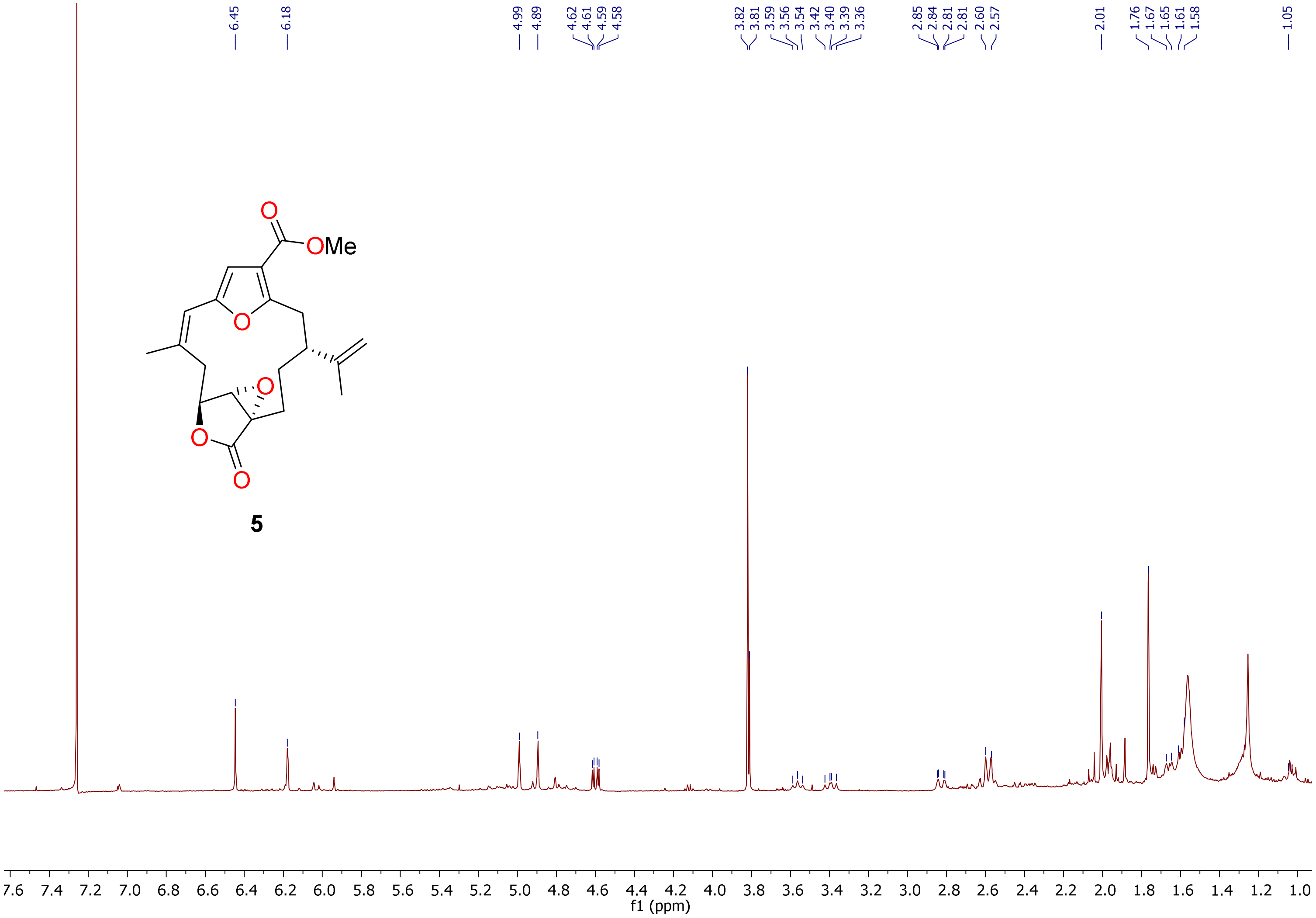

Figure 10. ^{13}C NMR spectrum of *Z*-isopukalide **5** in $CDCl_3$.

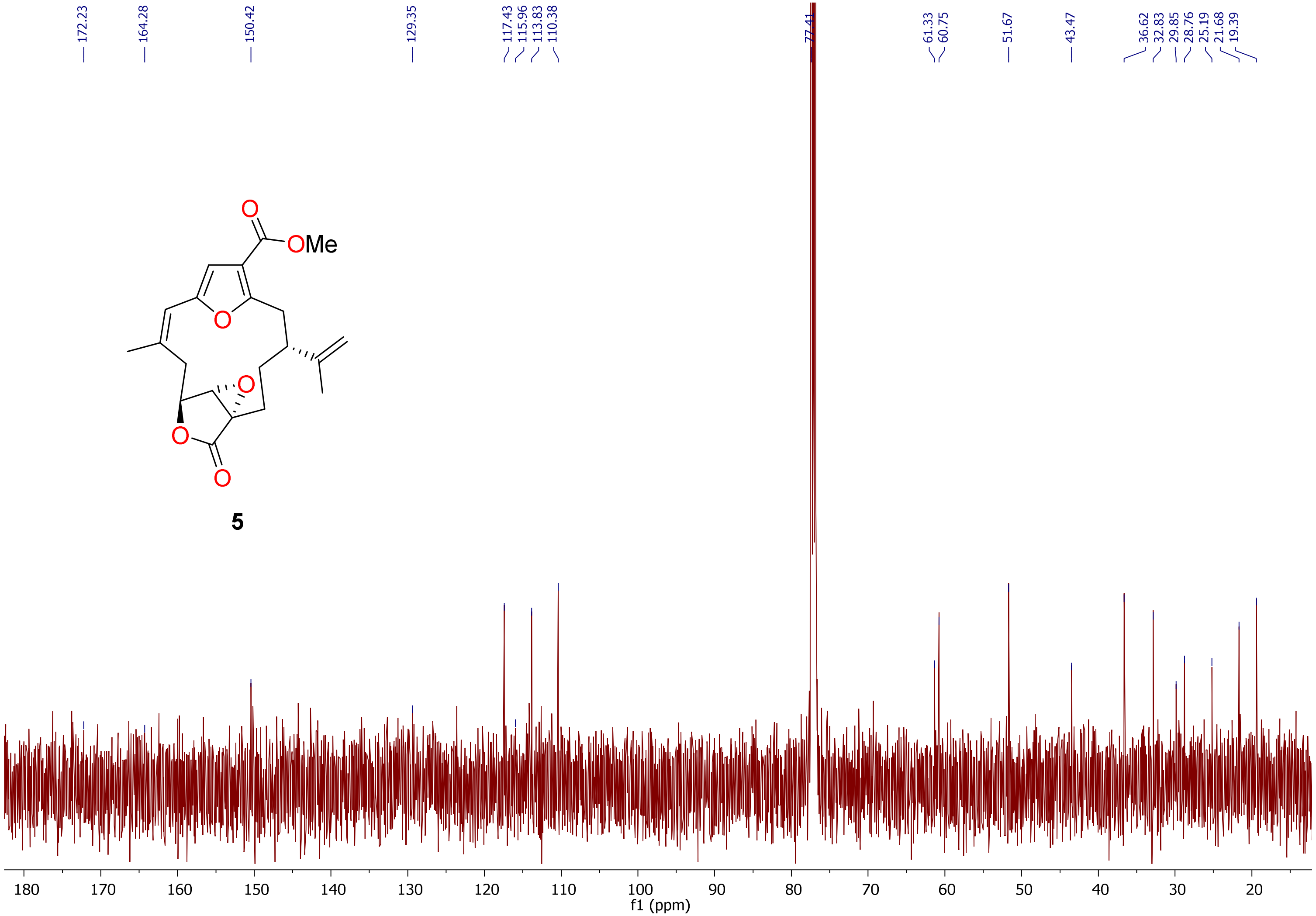

Figure S11. ^{1}H NMR of **6** in $CDCl_3$

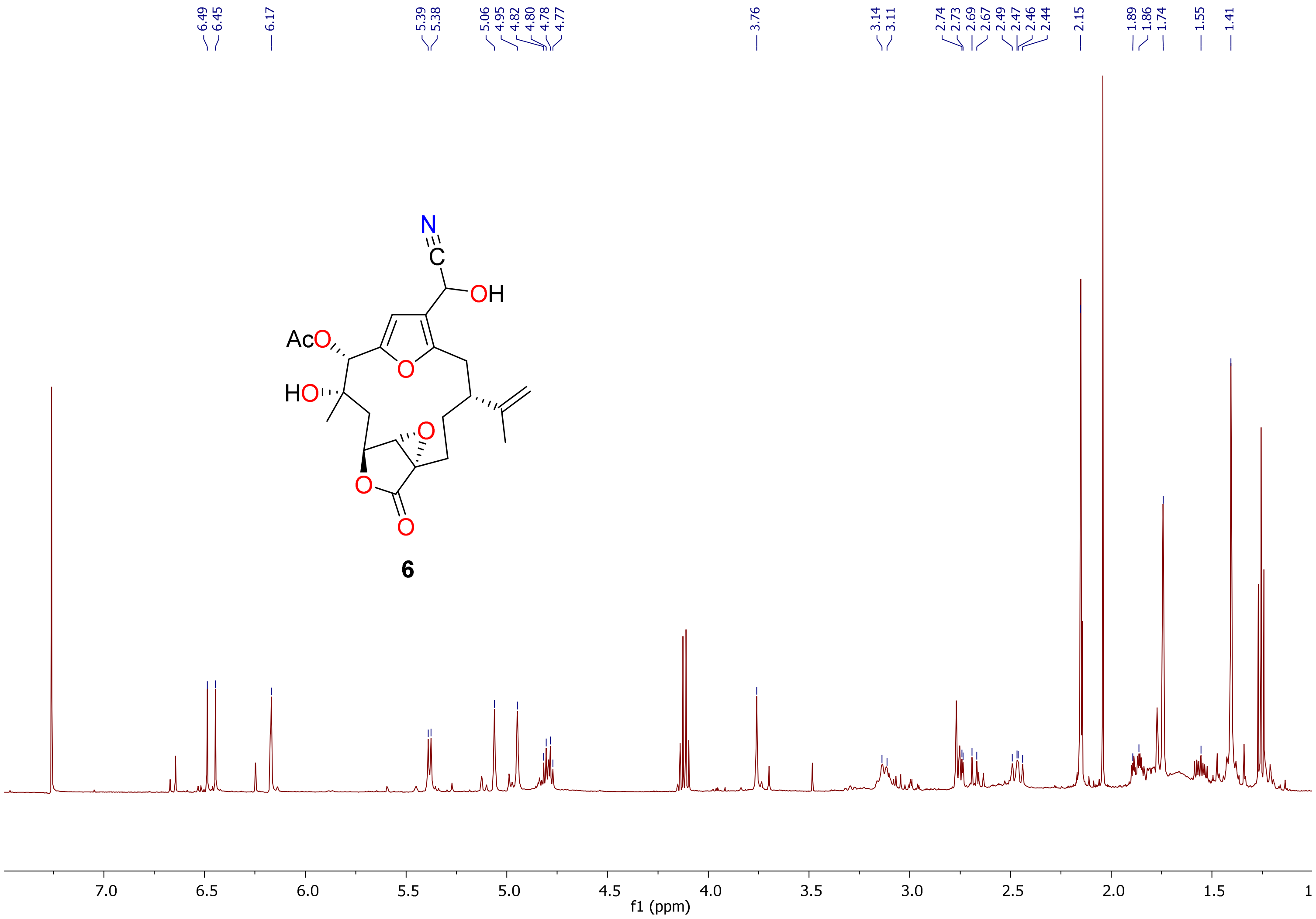

Figure S12. ^{13}C NMR spectrum of **6** in $CDCl_3$

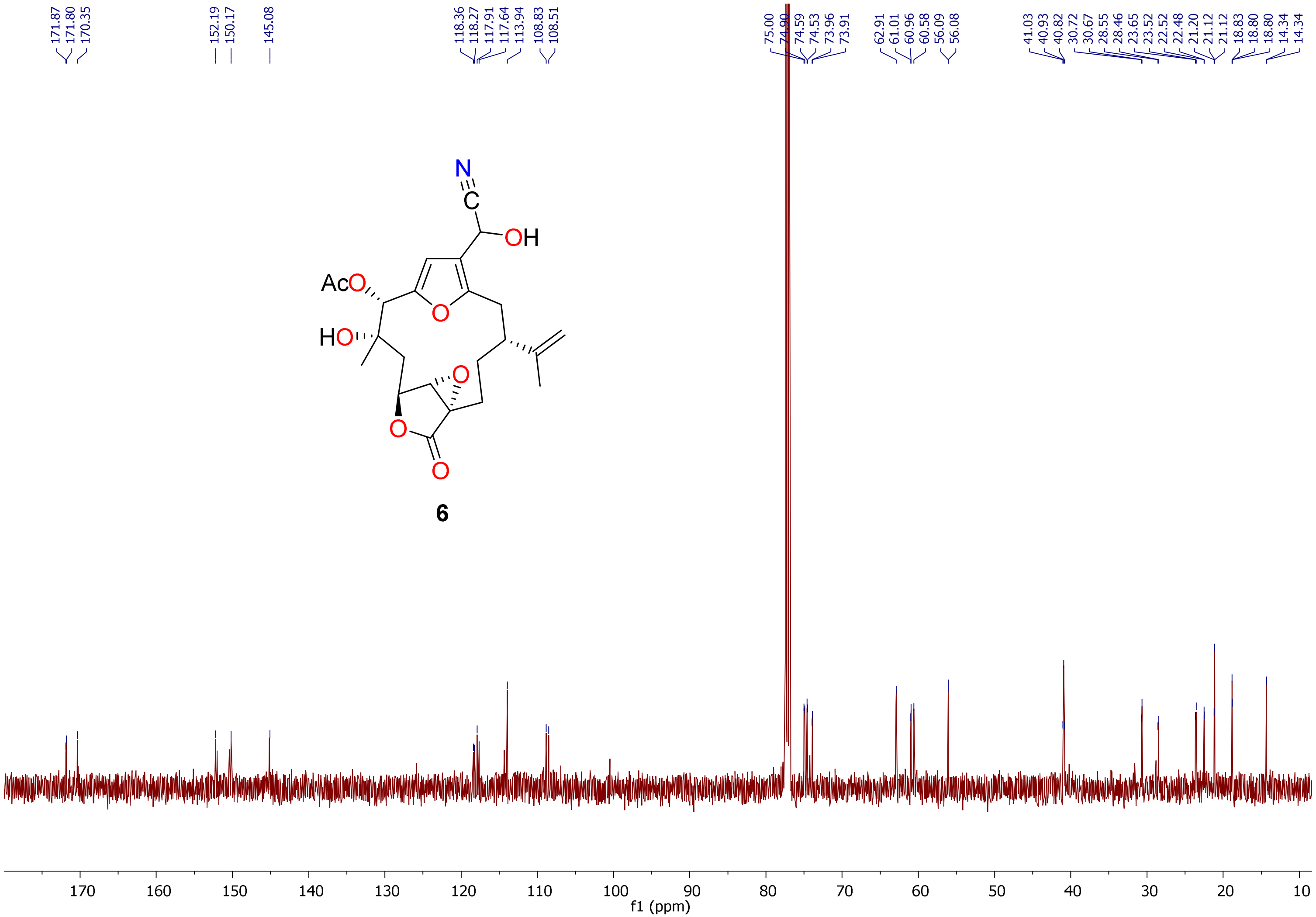

Figure 13. Minimized structures and selected NOE effects (↔) of 2, 4 and 6.

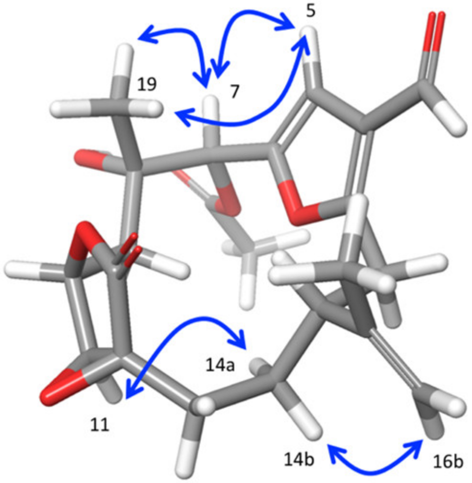

2

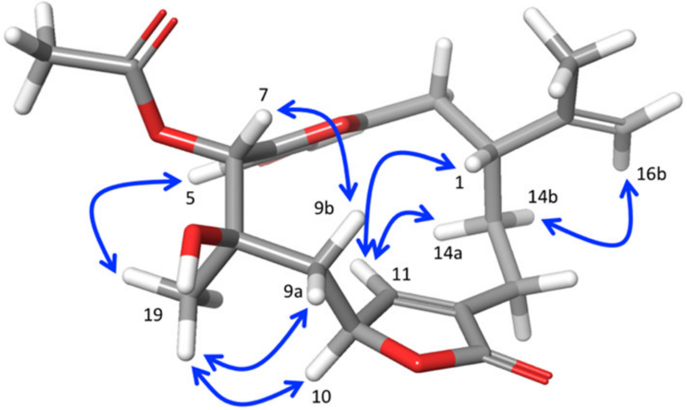

4

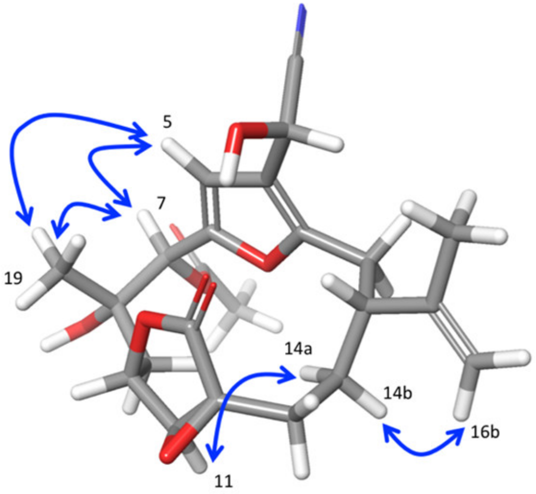

6

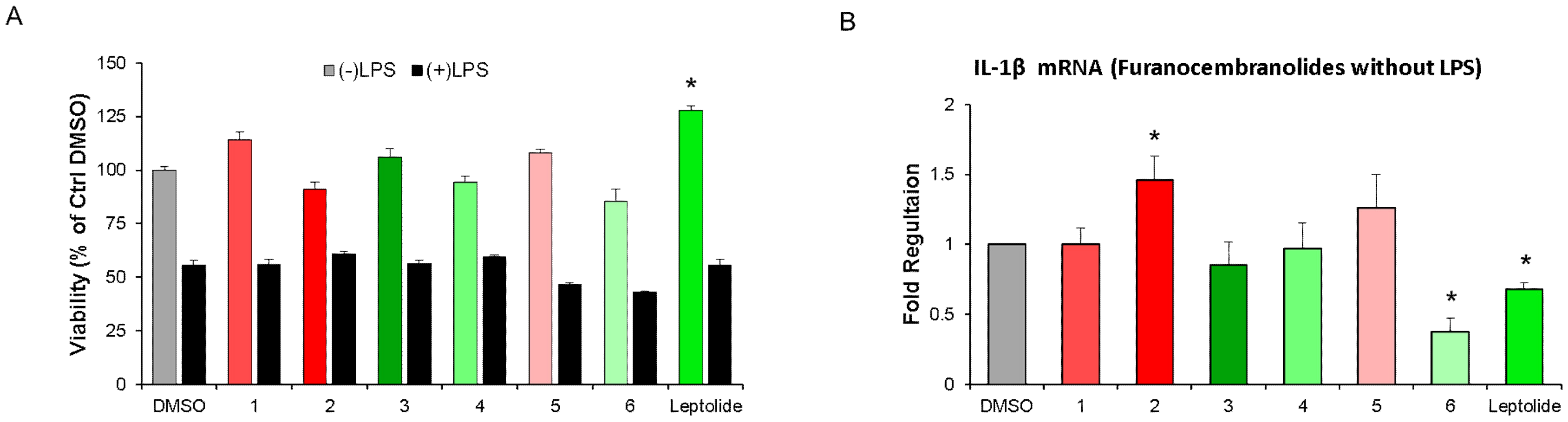

Figure S14. Effects of furanocembranolides on microglial cell viability and inflammation. A) Cell viability results measured by MTT, normalized to the Control DMSO group (termed as 100% viability). The bars show the mean value for quadruplicates, and error bars show the standard error mean. B) Total fold change of IL-1β mRNA production in BV2 cells treated only with furanocembranolides in the absence of LPS. Statistical differences were analyzed by one-way ANOVA followed by all pairwise multiple comparisons by Bonferroni t-test. Only biologically significant differences are shown.
